# Uncertainty-aware diabetic retinopathy detection using deep learning enhanced by Bayesian approaches

**DOI:** 10.1038/s41598-024-84478-x

**Published:** 2025-01-08

**Authors:** Mohsin Akram, Muhammad Adnan, Syed Farooq Ali, Jameel Ahmad, Amr Yousef, Tagrid Abdullah N. Alshalali, Zaffar Ahmed Shaikh

**Affiliations:** 1 Department of Computer Science, School of Systems and Technology, University of Management and Technology, Lahore, 54770 Pakistan; 2 Electrical Engineering Department, University of Business and Technology, Jeddah, 21448 Saudi Arabia; 3 Engineering Mathematics Department, Alexandria University, Alexandria, 21526 Egypt; 4 Department of Information Systems, College of Computer and Information Sciences, Princess Nourah bint Abdulrahman University, Riyadh, P.O. Box 84428, 11671 Saudi Arabia; 5 Department of Computer Science and Information Technology, Benazir Bhutto Shaheed University Lyari, Karachi, 75660 Pakistan; 6 School of Engineering, Ecole Polytechnique Fédérale de Lausanne, 1015 Lausanne, Switzerland

**Keywords:** Computational biology and bioinformatics, Medical research

## Abstract

Deep learning-based medical image analysis has shown strong potential in disease categorization, segmentation, detection, and even prediction. However, in high-stakes and complex domains like healthcare, the opaque nature of these models makes it challenging to trust predictions, particularly in uncertain cases. This sort of uncertainty can be crucial in medical image analysis; diabetic retinopathy is an example where even slight errors without an indication of confidence can have adverse impacts. Traditional deep learning models rely on single-point predictions, limiting their ability to provide uncertainty measures essential for robust clinical decision-making. To solve this issue, Bayesian approximation approaches have evolved and are gaining market traction. In this work, we implemented a transfer learning approach, building upon the DenseNet-121 convolutional neural network to detect diabetic retinopathy, followed by Bayesian extensions to the trained model. Bayesian approximation techniques, including Monte Carlo Dropout, Mean Field Variational Inference, and Deterministic Inference, were applied to represent the posterior predictive distribution, allowing us to evaluate uncertainty in model predictions. Our experiments on a combined dataset (APTOS 2019 + DDR) with pre-processed images showed that the Bayesian-augmented DenseNet-121 outperforms state-of-the-art models in test accuracy, achieving 97.68% for the Monte Carlo Dropout model, 94.23% for Mean Field Variational Inference, and 91.44% for the Deterministic model. We also measure how certain the predictions are, using an entropy and a standard deviation metric for each approach. We also evaluated the model using both AUC and accuracy scores at multiple data retention levels. In addition to overall performance boosts, these results highlight that Bayesian deep learning does not only improve classification accuracy in the detection of diabetic retinopathy but also reveals beneficial insights about how uncertainty estimation can help build more trustworthy clinical decision-making solutions.

Diabetic Retinopathy (DR) is a prevalent eye disease that primarily affects individuals with diabetes. It is a leading cause of blindness and visual impairment among people with diabetes, affecting millions of individuals worldwide. DR arises when the small blood vessels in the retina are damaged due to elevated blood sugar levels. Early detection and timely treatment can prevent or slow the progression of the disease, thereby preserving eyesight. A microvascular complication of diabetes, DR exemplifies the severe impact that diabetes can have on the body.

The progression of the disease follows various stages, beginning with non-proliferative diabetic retinopathy (NPDR), the milder form. As the condition advances, it can develop into proliferative diabetic retinopathy (PDR), a more severe stage characterized by the growth of abnormal blood vessels on the retina. These new vessels are fragile and can result in serious complications, including blindness, if not treated.

The stages of DR include:

- Normal DR
- Mild DR
- Moderate DR
- Severe DR
- Proliferative DR

Timely and accurate diagnosis of DR is crucial, as it assists ophthalmologists in providing effective care for patients with diabetes while also preventing complications associated with the disease. Prompt detection facilitates immediate medical treatments, thereby reducing the progression of the illness and lowering the risk of severe consequences. The impact of DR on eyesight underscores the importance of effective diagnostic methods and comprehensive care strategies. Figure 1 illustrates the connection between diabetes and DR. Addressing this issue effectively requires a multidisciplinary approach that includes healthcare professionals, advanced diagnostic technology, and patient education.

**Fig. 1:**
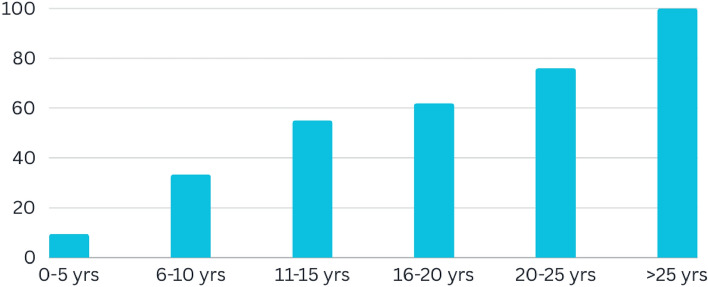
DR association with the duration of diabetes in patients.

Deep learning (DL) has significantly enhanced real-world applications including financial risk assessment, autonomous driving, and medical diagnostics. However, in domains such as medicine, full confidence in a model’s predictions is required before making any treatment decisions. DL models, in their current state, often lack uncertainty information in their predictions. However, limitations currently reduce the effectiveness of automated DR classification systems. While DL models achieve high accuracy in medical image analysis, they notably lack mechanisms for uncertainty estimation. The absence of uncertainty estimation is particularly concerning in clinical applications where reliable predictions are essential for decision-making. For cases that may need human intervention, such as borderline predictions or low-confidence cases, uncertainty quantification plays a key role. To address these issues, this study proposes a Bayesian convolutional neural network (BCNN) model based on the DenseNet-121 architecture, augmented with Bayesian approaches to provide uncertainty quantification in DR classification predictions. The model not only achieves high accuracy but also provides uncertainty estimates for each prediction through MC-Dropout and Mean Field Variational Inference (MFVI). These Bayesian methods allow the model to provide predictive entropy values and confidence scores, making it highly suitable for real-world clinical diagnosis where decision confidence is as critical as prediction accuracy.

Bayesian modeling provides a systematic framework for designing learning algorithms and training probabilistic neural networks. This concern is especially pronounced in fields such as medicine, where accurate detection of conditions such as DR is critical, and understanding uncertainty is crucial for relying on automated diagnoses. There is growing interest in developing DL models that are cautious about their level of certainty. Bayesian modeling is one of the most promising approaches in this regard. Bayesian deep learning (BDL) combines Bayesian inference principles with DL techniques to address the limitations of traditional DL models, particularly in assessing uncertainty. Unlike conventional DL models that produce point estimates, BDL incorporates uncertainty estimation by treating model parameters as probability distributions.

BDL involves Bayesian neural networks (BNNs), where weights and biases are not static but are instead treated as probability distributions. To integrate observed data for the estimation of the posterior distribution over parameters, BDL often employs Bayesian inference methods such as Markov Chain Monte Carlo (MCMC), MC Dropout, or MFVI. These methods allow for differentiation between model uncertainty (epistemic uncertainty) and data uncertainty (aleatoric uncertainty).

BDL aids in the improvement of decision-making by revealing uncertainty estimates which are indeed essential, especially for critical domains such as medical diagnosis^1^, brain tumor segmentation^2^ and breast cancer detection^3^. It enables physicians to check for the accuracy of predictions that will with time improve patient care and safety. Recent developments of robust classifiers also have gained much attention. The most popular is the approximate BDL approach. It provides an efficient approximation to the posterior distribution of BNN. In this respect, BDL could also help make personalized therapy a little closer to actuality for DR patients^4^.

These new data and uncertainty estimates can be incorporated directly into models to help provide more accurate predictions of what individual patients will receive across treatments. This is important in accuracy and robustness for applications such as DR diagnosis, and forecasting. The process also offers several medical benefits to the world of medicine, some include:

- BDL provides uncertainty estimations that allow doctors to make more informed decisions, especially when prediction performance is crucial.
- BDL can help to make AI-assisted medical diagnosis and treatment planning safer, and more reliable reducing the risk of making a bad judgment by quantifying uncertainty.
- BDL models are particularly relevant for medical research and personalized therapy, as they can learn from additional data to update uncertainty estimates.

The core contributions of this study are as follows:

1. *Development of Bayesian DenseNet-121 Model*: We fine-tuned a DenseNet-121 CNN model, trained using three different Bayesian approximation approaches, MC-Dropout, MFVI, and a deterministic baseline method. These approaches enable resilient classification of DR as well as uncertainty estimation based on retinal images.
2. *Predictive Uncertainty for Clinical Applicability*: This study improves model reliability by quantifying predictive uncertainty using Bayesian models, providing insights critical in a clinical diagnostic setting. The model’s predictive entropy and accuracy metrics help identify cases with appropriate levels of uncertainty, supporting medical decision-making.
3. *Comparative Evaluation and High Accuracy*: The model’s efficacy as a potential clinical diagnostic tool is supported by high classification accuracy and reliable performance in comparative evaluations against existing DR classification methods, with the proposed model showing significantly improved results. These contributions help relate the model to medical image analysis and enhance its applicability in clinical settings for improving DR diagnosis.

## Organization of paper

The paper is organized as follows: the “Literature review” section highlights the literature review, including related research on DL, transfer learning, BDL, and DR. The “Materials and methods” section elaborates on the dataset, study, and experimental methodology. In the “Results and discussion” section, the experimental data and results are presented. Finally, the “Conclusions and future work” section summarizes the findings derived from the results along with future research and potential advancements in this field.

## Literature review

In recent years, significant scientific work has focused on the applications of DL and models that quantify uncertainty in its predictions. This section reviews the latest developments in this area.

### DR detection using deep learning

Mary and Kavitha^5^ developed a DL-based approach for DR detection by combining DenseNet-121 and ResNet-50 architectures with Shapley Additive Explanations, offering an ensemble model that improves interpretable detection performance (SAE-DR). The dataset employed in the study contained fundus images from the APTOS 2019 Dataset and data augmentation techniques were applied (rescaling, flipping, rotating, and zooming) to mitigate the scarcity of available data and make the model more robust. After augmentation, images are pre-processed to enhance features of interest and then are subsequently segmented with a revised U-Net model allowing great spatial resolution for retinal blood vessel delineation. The explained ensembled DenseNet-121 and ResNet-50 for DR severity classification based on retinal features such as microaneurysms, exudates, and hemorrhages accurately detect the DR severity. Compared to other detection and interpretability quality state-of-the-art methods, the model provided 98.69% accuracy (sensitivity = 86.23%, specificity = 97.54%, F score:90.26% precision:94.26%; Processed in: 0.153 sec).

Yang et al.^6^ proposes an improved systemic way to detect DR, which is based on a model formed by joining the Inception-V4 neural network with a more efficient dynamic Snow Leopard Optimization (DSLO) that can be used to select features and predict results. The improved features selection by DSLO allowed to Inception-V4 model to promote DR early signs like leaking vessels and optic nerve edemas as abnormalities with better detection. The Inception-V4/DSLO model was compared to several best existing models on the DR 2015 dataset, namely MLLD, PCNN/ELM, DRFEC, R-AlexNet and Deep-DR. The results indicated that Inception-V4/DSLO could appropriately detect early stages of DR cases in comparison to conventional ML and DL models, which shows its competency for computer-aided DR screening.

Patil et al.^7^ overcomes some multi-class classification challenges like accuracy and data imbalance. They leverage the concept of transfer learning by training a ResNet-50 model on the APTOS data set and testing on EyePACS, thus achieving better generalization performance over the unseen portion of data with results going beyond classification. Regarding this, the authors did a lot of image pre-processing and augmentation which they seemed tuned based on experience as it worked better. TTA: Test Time Augmentation (TTA) was used as well to set up some better results for model performance over layer. Finalizing the model achieves a high multi-class accuracy of 97.87% and a Quadratic Weighted Kappa (QWK) score of 0.985 indicating its effectiveness in DR classification and at the same time, wind up systematic data augmentation effectively to boost the performance capabilities of the model for robustness aspect.

Lin and Wu.^8^ propose DR detection with the application of visualization, and pre-processing methods to improve a DL model based on ResNet-50 performance. The study achieves this by updating using ResNet-50 architecture with adaptive learning rates, layer-weight adjustments, and regularization to tackle overfitting and loss value fluctuation. The modified ResNet-50 outperformed the other CNN models, Xception, AlexNet, VGGNet-s, and VGGNet-16 with a training accuracy of 0.8395 and a test accuracy of 0.7432. These performance increases indicate that the modifications adequately mitigate overfitting and/or increase stability during training, specifically with respect to model convergence. Amongst our contributions in this study are a Standard Operating Procedure SOP for the pre-processing of fundus images and visual insights as to how the optimally revised ResNet-50 can then be modified to maximize model calibration at the expense of screening performance This work is an example of how CNN structural adjustments may influence DR grading performance and serves as a basis to further refine CNN structures in medical imaging^9^.

Mutawa et al.^10^ using transfer learning techniques perform identification of DR images to understand the performance of various models through multiple datasets using CNN architectures. In particular, four CNN-based transfer learning models i.e. VGG16, InceptionV3, DenseNet121, and MobileNetV2 are used in the study and their performances were analyzed on three different datasets containing retinal images. Loss, accuracy, recall, precision, and specificity were some of the key metrics evaluated. Given the variability of retinal features across datasets, the authors further evaluated model performance on a merged dataset of images from all three sources. DenseNet121 was found to be the best model giving a 98.97% accuracy on the combined dataset, demonstrating that pooling data from various sources will perform better on classification than using single-dataset approaches. This model has a promise for wider clinical application as it provides clinicians with a tool for early DR risk prediction and referral of patients to ophthalmologists to avoid harmful sequelae of severe DR.

Islam et al.^11^ propose a new method for the detection and severity classification of DR using supervised contrastive learning (SCL) along with the Xception CNN model. Entry-level DL models are often used with cross-entropy loss, which can be susceptible to noise and hyperparameters; SCL delivers a robust two-stage training contrastive loss function that improves margin and classification accuracy. It improves the image with contrast-limited Adaptive Histogram Equalization (CLAHE) and uses transfer Learning which takes Xception as the encoder. The t-SNE method is used to visualize the data points in 2D space, helping us interpret the embedding space learned by SCL in real time. The model was tested on APTOS 2019 and Messidor-2 datasets, producing a 98.36% accuracy of test data with an AUC of 98.50% for binary classification and values of 84.36% with an AUC of 93.82% for grading DR severity over five stages. SCL achieves significant accuracy improvement for DR detection from complex medical images over the traditional CNN models without SCL.

Nunna and Tiwari^12^ propose the development of a DL method with an embedded attention mechanism to automatically detect DR, improving both diagnostic throughput and accuracy over manual approaches. This way of doing it took CNNs with a self-attention module (from transformer networks) to find great combinations of feature maps extracted from the different ResNet and DenseNet architectures. This self-attention mechanism enables the model to better contextualize features from these networks, further enhancing its ability to identify lesions and grade DR severity on fundus images. The model was validated on a dataset for the classification of DR; the test results achieved an accuracy of 89.09% showing potential for efficient DR screening. This approach describes how you can leverage attention mechanisms to improve the model performance in complex medical image classification.

Yi et al.^13^ introduced a new diagnostic performance model, RAEfficientNet, for DR grading. This model combined residual attention blocks with EfficientNet for improved feature extraction and was implemented on the APTOS 2019 dataset. RAEfficientNet was used for DR classification into 2-grade and 5-grade levels, showing significant performance improvement with a 5-grade classification accuracy of 93.55% and a 2-grade classification accuracy of 98.36%. These findings suggest that advanced DL architectures and attention mechanisms can greatly enhance a DR detection model’s precision.

Alyoubi et al.^14^ addressed the problem of DL model development for DR diagnosis. They trained the classical CNN512 model on tagged retina images representing different DR stages. The second DL model was designed for DR lesion localization, using the updated YOLOv3 architecture. Despite YOLOv3’s poor mean average accuracy of 0.216 on the DDR dataset, merging CNN512 with YOLOv3 allowed the categorization of DR pictures and identification of lesion coordinates with 89% mean accuracy, 89% sensitivity, and 97.3% specificity.

### DR detection using hybrid learning

Jabbar et al.^15^ proposed for detecting DR and extended the previous lesion-based methods which only provide detection of early or severe-stage lesions. A Robust Features Extraction Model based on GoogleNet, ResNet, and APSO. After features have been extracted, they are classified by random forest, support vector machine (SVM), decision tree, and linear regression models. While previous models have primarily focused on early lesions such as exudates and hemorrhages, this method also includes severe lesions like cotton wool spots, venous beading, and RPE damage. Using a benchmark dataset the hybrid framework yielded an accuracy of 94% and achieved significant improvements in precision, recall, accuracy, and F1 score at different DR severity levels. This approach demonstrates the potential utility of a multi-lesion strategy for accurate diagnosis of DR.

Taifa et al.^16^ proposed a hybrid solution that combines machine learning classifiers and DL feature extractors for DR detection and classification. They exploit the use of MobileNetV2, DenseNet121, and InceptionResNetV2 as feature extractors to retinal images and aggregate their outputs with a stacking ensemble framework combining two machine learning classifiers such as Decision Trees, Random Forests, and SVM. Hyper-parameter tuning uses Random Search and Grid Search to optimize performance. The APTOS 2019 Blindness Detection dataset was used for testing on this hybrid model achieving a multi-class classification accuracy of 95.50% and binary class base classification accuracy of 98.36% with DenseNet121 giving the best performance as extractor alone. This is a good step forward to achieve reliable detection of DR at earlier stages, which is crucial for timely treatment.

Thomas and Jerome^17^ proposed an ensemble model that classifies DR, successfully using transfer learning coupled with an SVM classifier for increased accuracy. On fundus images obtained from publicly available datasets (Messidor, EyePACS) and an in-house clinical dataset, the proposed approach first applies a Trilateral Filter for noise reduction followed by contrast-limited Adaptive Histogram Equalization with an unsharp mask to enhance the contrast. After the segmentation of both enhanced images, the segmentation uses Extended Piecewise Fuzzy C-Means Clustering (EPFCMC) to segment the thick blood vessels. GLCM features are extracted and selected from these segmented images through a War strategy optimization algorithm. Finally, an ensemble of 3 CNN models (called thrice CNN) is fused with an SVM classifier giving a high accuracy of 98.94% in differentiating DR from normal cases. This model is reliable in DR classification and may assist clinicians in diagnosing DR at an early stage.

Mohanty et al.^18^ investigate the detection and classification of DR with two models, a VGG16-based hybrid network combined with an XGBoost classifier, and DenseNet 121. However, to tackle the class imbalance present in the APTOS 2019 Blindness Detection Kaggle Dataset pre-processing and data balance methods were utilized. The hybrid VGG16-XGBoost model obtained 79.50% accuracy in testing and was strongly outperformed by DenseNet 121 which achieved 97.30% accuracy. These better results highlight how DenseNet 121 is effective when it comes to automated DR detection. These results indicate that DL techniques, with specific emphasis on DenseNet 121 architecture, have the potential to increase both efficiency and accuracy of diagnosis which may translate into faster and more accurate screening of DR for the healthcare providers^19^.

### DR detection with uncertainty quantification

Verma et al.^20^ introduce an uncertainty-aware CNN model for DR detection that alleviates the well-known problem of overconfident incorrect predictions in most medical DL models. The model makes extensive use of train-time data augmentation for better generalization and incorporates multiple probability calibration techniques including Platt scaling, temperature scaling, isotonic regression histogram binning, and Platt histograms to produce reliable probabilities. Then, TTAUG (Test Time Augmentation) increases the accuracy of predictions by re-evaluating the images through drug transformations. Compared to the 93% accuracy in Kaggle without TTAUG, the model had around 5 percent higher accuracy (about 98%) with TTAUG on the Kaggle dataset and we evaluated it on the Messidor dataset also. Further, the application of calibration decreased the Expected Calibration Error (ECE) from 0.52 to 0.019, underlining how essential calibrated probability estimates for interpretation and diagnostic purposes. This shows that well-calibrated uncertainty-aware models can increase both the accuracy as well as trust in automated DR detection.

Siebert et al.^21^ proposed the integration of EfficientNet-B0 architecture within a Deep Kernel Learning (DKL) model by leveraging the theoretical benefits of GP for uncertainty estimation. The model aims to improve automated DR screening by flagging highly uncertain cases for specialist review automatically. It includes new recent extensions to the DKL framework that enhance miscalibrated uncertainties and demonstrate its efficacy at finding near out-of-distribution (OOD) samples, such as images of other eye diseases through high epistemic uncertainty. In addition, the DKL model refines aleatoric uncertainty calibration across DR-related datasets while boosting confidence in diagnosis. The study findings indicate that this method can enhance safety for DR screening and reduce the risk of missing other significant non-DR pathologies by accurately identifying uncertain cases.

Hassan and Ismail^22^ propose BDL approaches for improving DR detection, yet emphasize the need for uncertainty quantification in model predictions in this paper. The authors design CNN architecture for DR classification and add two Bayesian Extensions - Variational Inference (VI) and Monte Carlo Dropout (MC-Dropout) allowing the increase of confidence in predictions from the model. Two models achieved 94.4% test accuracy by CNN without uncertainty, and a similar performance using VI (94% test accuracy) and only slightly lower the accuracy with dropout-based Monte Carlo approximations (93.3%). on the 2019 APTOS dataset The methods proposed, based on the entropy and standard deviation of the posterior predictive distribution estimate cleanly model uncertainty and alleviate issues caused by overconfident predictions from conventional DL models.

Jaskari et al.^23^ investigate whether DR classification using uncertainty-aware DL is more robust than non-uncertain methods by proposing BNNs which have a considerable advantage of providing accurate estimates of uncertainty in the underlying data compared to plain neural networks. Firstly, it studies a two-class problem (referable/non-referable DR) and a more complex 5-class classification of DR severity, validating nine BNNs on clinical as well as public benchmark datasets. Thus, this work demonstrates that traditional entropy-based uncertainty measures, which tend to yield better binary classification results for several well-established benchmark datasets, do not automatically imply improved performance over a clinical dataset employing the 5-class classification scheme. Finally, they show that a new measure of uncertainty motivated by the link between entropy-based uncertainty and classifier risk is more generalizable when evaluated on clinical data, leading to improved robustness against data. We conclude from this work that, even if benchmark-optimal uncertainty methods have been successfully developed in research contexts, these are not necessarily the best choices for clinical settings, nor are direct transfers possible.

Band et al.^24^ proposed benchmark competitions for the diagnosis of DR using BDL. They evaluated many Bayesian and non-Bayesian models for forecasting tasks according to different task-specific criteria or forecast hypothesis measures. The study used two large retinal datasets (APTOS and EyePACS) to investigate different Bayesian inference strategies at scale, including Structured Mean Field Variational Inference (SFVI), MC-Dropout, MFVI, maximum a posteriori estimate (MAP), and deep ensembles. The strength of this paper is its systematic assessment of several BDL models across many datasets. The authors supported experimental results by quoting implementations of multiple benchmark methods on widely distributed significant computational resources-e.g., 20-GPU days, 100-TPU days, and selection over about 400 hyper-parameter combinations.

From the related studies mentioned above, while individual methods of DR diagnosis with DL models have shown promising results, their clinical use often relies heavily on these model predictions, which may not always be fully trusted due to confidence gaps in DL model predictions. Additionally, studies that propose uncertainty quantification for DL models can measure model reliability, but their dependability is limited by the need for large, real-world datasets to train these models from scratch. Also due to the availability of large annotated datasets and the need for significant computational resources, training of these models from scratch is not a reliable approach. Transfer learning has emerged as a useful way to address these limits in medical image analysis. However, limited research has applied transfer learning to improve DR diagnosis, and areas like model uncertainty quantification need further exploration to boost the reliability of DR diagnosis.

Our proposed work aims to overcome the challenges of DR detection using a transfer learning-based framework that can leverage pre-trained DL models to improve the accuracy of DR detection while addressing the limitations of uncertainty quantification of previous methods using Bayesian approximation methods.

## Material and methods

This study established a novel multi-layer architecture that used the pre-trained DenseNet121 CNN, combined with Bayesian modeling for DR classification based on retinal images. The suggested approach involves the following major stages:

1. *Pre-Processing of Retinal Images*: Retinal images are pre-processed to gain contrast enhancement. This stage is aimed at enhancing and improving the quality of retinal images. This enhancement is targeted at highlighting important retinal features.
2. *DenseNet-121 CNN & Bayesian Modeling*: The DenseNet-121 pre-trained CNNs are then fine-tuned and trained using Bayesian modeling with approximation methods like MC dropout, MFVI, and Deterministic for posterior predictive distribution.
3. *Uncertainty Quantification of BCNN Models*: Compute Uncertainty from the posterior predictive distribution using BCNN models via different matrices such as predictive entropy, AUC, and model accuracy based on posterior predictive distribution. This step is very crucial in medical diagnosis because it provides information about the accuracy of the model predictions. That probability is quantified through a predictive distribution, which captures uncertainty in the parameters of our model^25^.

**Fig. 2:**
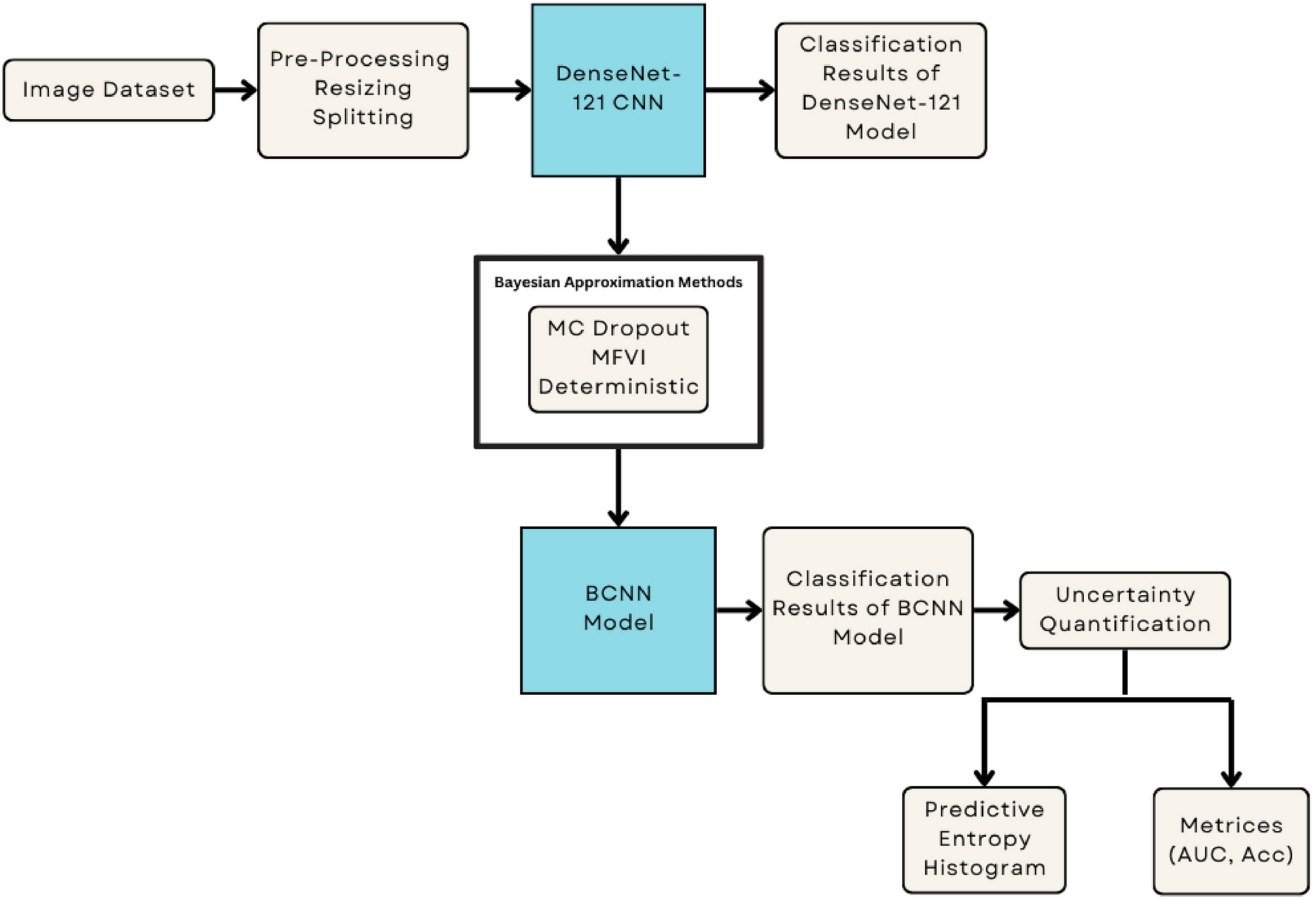
Flow diagram of the proposed methodology.

Figure 2 shows the proposed research methodology diagram.

### Dataset used

Our study utilizes two different Kaggle datasets. The first dataset is APTOS-2019, donated by the Asia Pacific Tele-Ophthalmology Society^26^. This dataset is one of the most widely used datasets currently available for detecting DR.

The APTOS-2019 dataset contains 3,662 color retinal images from different clinical settings. The image sizes range from 640$\times$480 to 2,848$\times$4,288 pixels. The grades had been rated at the DR severity scale from 0 to 4: No-DR, Mild-DR, Moderate-DR, Severe-DR, and Proliferative-DR, with 1,805 photographs in No-DR, 370 in Mild-DR, 999 in Moderate-DR, 193 in Severe-DR, and 293 in Proliferative-DR.

**Fig. 3:**
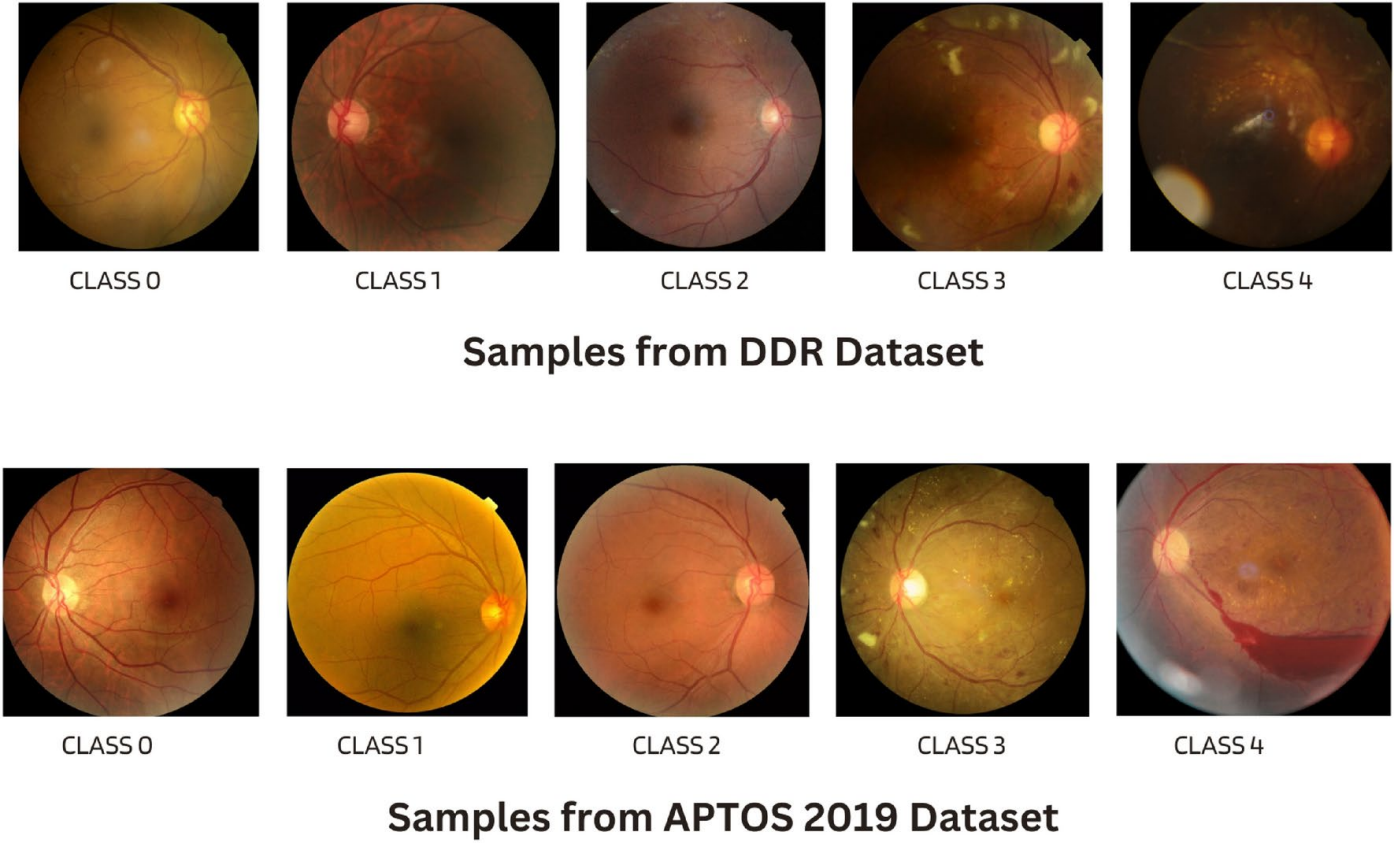
Labels of DR in the APTOS-2019 and DDR datasets.

The second one is the DDR dataset^27^, which is composed of 13,673 fundus images retrieved from all provinces (147 hospitals). This dataset is also separated into five groups of DR based on their severity. Both datasets contain severely skewed fractions with a significant amount of noise, hence requiring distinct pre-processing procedures to prepare the images for model training^28^. The main aim is to build a strong robust model which delivers adequate performance even when there are noise and modifications in the image. Figure 3 presents sample images from APTOS-2019 and DDR datasets for DR categories at various severity levels.

### DenseNet-121 CNN model

**Fig. 4:**
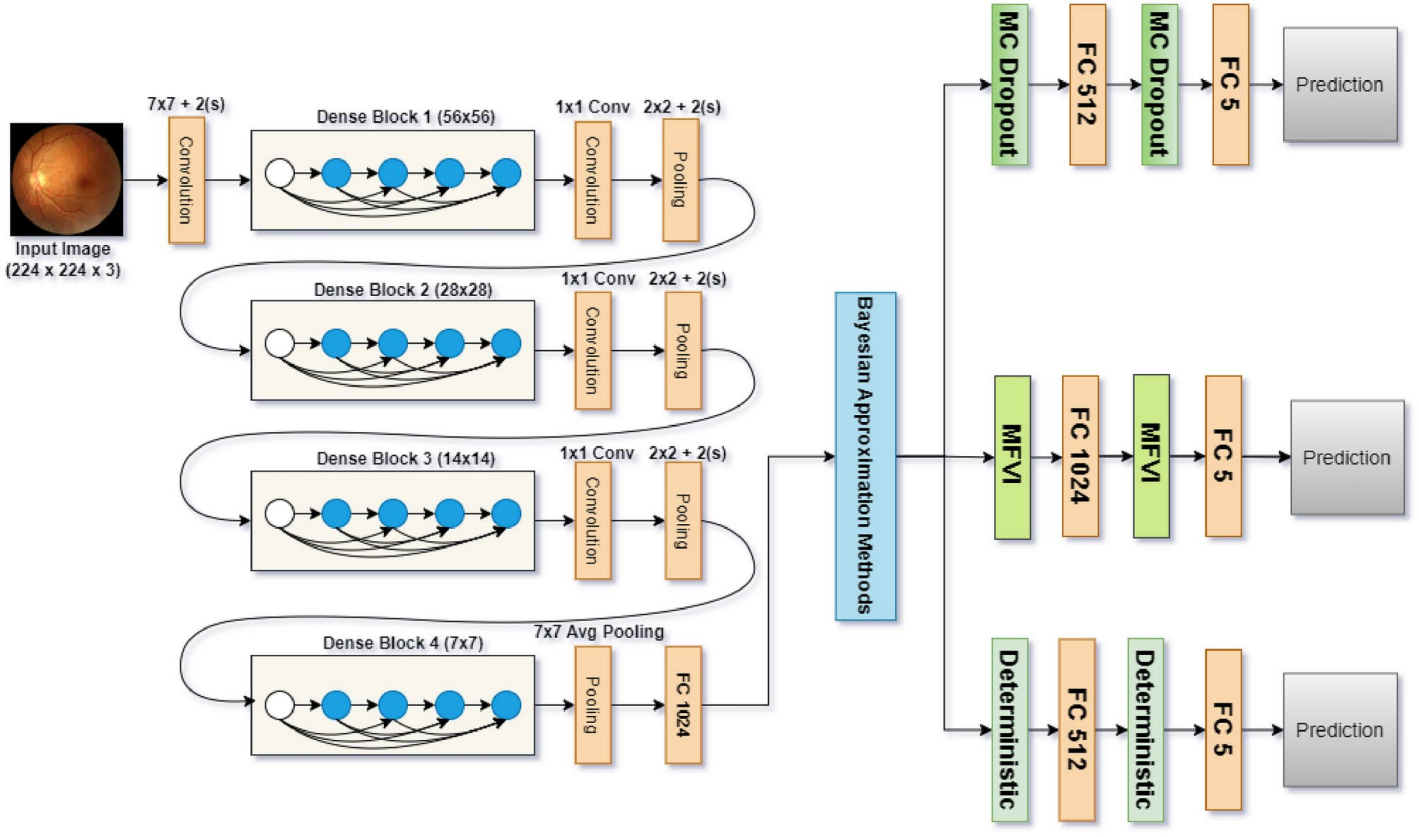
Architecture diagram for the proposed Bayesian DenseNet-121 CNN model.

DenseNet CNN has a pattern where each layer is connected to every other in a feed-forward manner^29^. This enables networks to grow deeper. This is the connectivity structure known in DenseNet as every layer takes input from all its preceding layers and passes on their feature maps to subsequent levels. The dense connection facilitates direct information flow across the network and supports feature reuse throughout the (layer stack). Figure 4 illustrates the design of our model of Bayesian DenseNet-121.

DenseNet architecture basics are convolutional and pooling layers, dense blocks, and transition layers. First, we have a convolutional block that cascades and applies a 7$\times$7 filter to the original image, resulting in 64 feature maps. This block applies a stride of 2 the spatial resolution decreases due to the down-sampling feature map. Another max-pooling layer with 3x3 filter and stride to further decrease the spatial dimensions in feature map^30^.

The DenseNet architecture is composed of dense blocks, which are followed by an operation block. A convolutional block in a Dense Block begins with batch normalization to normalize the input and ends with Rectified Linear Unit (ReLU) activation as nonlinearity. Then comes a 2D convolutional layer (or 2D Convolution) layer where the convolution takes place. In contrast, the series is repeated five times in the first dense block from DenseNet-121 and then it continues with one Densely connected model based generating a repeat of 12 and 24 times aside on the third dense block followed by another 6 repetitions for the fourth at last stage^31^.

In the DenseNet architecture, we have transition layers to reduce the number of channels in feature maps. These layers are used after each dense block and they consist of a 1x1 Convolution followed by a 2x2 average pooling with stride S=2. The number of channels is gradually dropped as features are put through this configuration dense block by dense block. The 256 channels of DenseNet-121 are reduced to 128, then to only 64, and finally even down to only the number necessary for output classification. Counterparts compared in this paper can be found on Kaggle.

Channel-wise pooling is performed at the end of those dense blocks and concatenated to provide a fixed-length representation similar to Global average pooling to wrap up all (final). This operation results in fewer feature maps that carry the most necessary information. A fully connected layer (dense) is used in a classification head with softmax activation for output class probabilities. The architecture of DenseNet can be seen from the description, shown in Table 1.

**Table 1:** Details of DenseNet-121 CNN Architecture.

Layers	Output size	DenseNet-121
Convolution	112x112	7x7 Conv, stride 2
Pooling	56x56	3x3 Max Pooling, stride 2
Dense Block (1)	56x56	[1x1 conv, 3x3 conv] x 6
Transition Layer (1)	56x56	[1x1 conv], 2x2 Avg Pool, stride 2
Dense Block (2)	28x28	[1x1 conv, 3x3 conv] x 12
Transition Layer (2)	28x28	[1x1 conv], 2x2 Avg Pool, stride 2
Dense Block (3)	14x14	[1x1 conv, 3x3 conv] x 24
Transition Layer (3)	14x14	[1x1 conv], 2x2 Avg Pool, stride 2
Dense Block (4)	7x7	[1x1 conv, 3x3 conv] x 16
Classification Layers	1x1	7x7 Global Avg Pool, FC Layer, Bayesian method Layers, Softmax

### Bayesian modeling

To account for the prediction uncertainty in the model, we transform the DenseNet-121 architecture to a Bayesian dense block convolutional neural network (CNN). While traditional CNNs treat the weights and biases as deterministic variables, with a Bayesian CNN we handle these probabilistically. The Bayesian CNN uses this method to efficiently exploit the dataset variability and provide a reliable estimation of uncertainty in its predictions^32^. For the weights and biases, we put a prior distribution on these by assigning them to the Bayes method for generating their posterior distribution. This posterior distribution is then used for generating a number of weight sets with which an ensemble of networks is trained. Ensemble networks are used to average the output of these ensemble networks and provide a probability distribution for each weight^25^.

In an ANN model with $L$ layers, such as a CNN, the weights affecting the model are introduced by a vector ${\textbf{w}} = \{ w_i \}_{i=1}^L$. In the context of Bayesian fitting, one estimates uncertainty over network parameters $P({\textbf{w}} \mid {\mathcal {D}})$ with respect to a dataset ${\mathcal {D}}$ (given some prior distribution on neural network weights $P({\textbf{w}})$). This prior distribution encodes beliefs about what neural network parameters are most reasonable for generating that data. At inference, we compute the predictive probability of our model $y$ for a new test input ${\textbf{x}}^*$ by integrating out all ${\textbf{w}}$ values^33^.

1 $$\begin{aligned} p(y|{\textbf{x}}^*,{\mathcal {D}}) = \int _{{\textbf{w}}} p(y|{\textbf{x}}^*,{\textbf{w}})p({\textbf{w}}|{\mathcal {D}})d{\textbf{w}} \end{aligned}$$

However, the true expression of this integral in real settings is given by Eq. 6. Equation 1 is usually computationally prohibitive because the likelihood function $P({\textbf{w}}|{\mathcal {D}})$ is difficult to compute. Consequently, a multitude of approximation strategies have been suggested to enable analytically solvable inference. Expectation propagation method, MCMC probability sampling-based approximation inference, MC-Dropout approximations, and MFVI approximations. Thus, these approximation techniques enable you to build the probability distribution for network parameters and infer how uncertain in practice our posterior is using approximations instead of calculating the expected value of a constant C derived by replacing the right side variable with calculated uncertainty as an integrated denominator. With our DenseNet-121 CNN model we use most of the approximation strategies in this approach see some methods below which show how to work on this^34^.

#### BCNN-MC dropout

MC dropout provides a DL-based approach to quantify the uncertainty in model predictions. It generalizes the popular dropout regularization technique for neural networks. In a conventional dropout setting, during the training some neurons are randomly dropped out with probability $p$ which helps the model to learn good features and prevent it from overfitting. At inference time, all the neurons are activated making feedforward-based predictions of test samples deterministic.

The approach is further extended using the Bayesian MC dropout method, which treats the dropout rate $p$ as being drawn from a probabilistic distribution. This enables uncertainty estimation in weight access through Bayesian inference against model weights. During inference, the model stochasticizes the predictions, meaning that some units are deterministically dropped out, allowing for uncertainty estimation.

This results in an approximate prediction distribution, allowing us to get some kind of measure for model uncertainty^25^. This technique automatically ensures every neuron contributes to the prediction process without overfitting. Recent work has shown that the use of dropout for regularization in deep networks corresponds to an approximate variational Bayesian inference. The key idea is to use dropout regularization during training and perform dropout (Bernoulli) sampling during testing. It has been shown that combining the mean-weight matrix (L-layered NN) and dropout probabilities (variational parameters) for a dropout distribution follows $\theta = \{M_l, p_l\}_{l=1}^L$, where $q_{M_l}(w_l) = M_l \cdot [\text {Bernoulli}(1 - p_l)]$. This can be viewed as the variational distribution $q_{\theta } (W)$, with the likelihood $p(y=c|x, {\hat{W}})$. Consequently, multiple stochastic forward passes (Monte Carlo sampling) produce $T$ instances of ${\hat{W}} \sim q_{\theta } (W)$, resulting in ${\hat{p}}(y=c|x, {\hat{W}}) = \frac{1}{T} \sum _{t=1}^T {\hat{p}}(y=c|x, {\hat{W}}_t)$.

#### BCNN-MFVI

MFVI is the second method used for inference, and it may be known by many as a key Bayesian tool to derive estimates of complex posterior distributions. In a nutshell, the concept behind MFVI is to approximate joint posterior probability distribution through factors (m number of factorized distributions). As a result, factorization can give rise to efficient computation and inference in BNN. This method uses a neural network in which some parameters (weights and biases) are modeled as some specific predefined prior distributions^32^. The basic principle is to infer a more precise posterior probability distribution of these variables from the real data.

In our implementation of the BCNN-MFVI model, we ultimately chose a Gaussian prior means and moderate standard deviation to balance model complexity that addresses the overfitting problem. This choice was important to adequately learn model uncertainty and training robust features. To minimize the gap between the two, MFVI uses a Kullback-Leibler Divergence (KLD) as an illustrative loss function to control how much these approximations are actually similar. Similar techniques are used to address the computational cost and instability in gradient estimations like the reparameterization technique and Flipout MC estimator. We use MFVI due to iterative optimization and its scalability, which is needed for the high dimensionality of BNN. Specifically, this approach includes probabilistic measurements of the degree of uncertainty in predicting model output and more general information on reliability (model prediction dependability) and robustness (learnt representations resilience).

#### BCNN-deterministic

Besides the bayesian approximation, our model employs (softmax) deterministic techniques for prediction problems. Deterministic models are characterized as calculating model parameters’ point estimates and producing definitive predictions without quantifying uncertainty. Deterministic models convert raw scores into probabilities using the softmax activation function in the final layer. Softmax would normalize the output logits so that they can be interpreted as predicted probability, adding up to one for each class. Our model incorporates both deterministic and Bayesian approximation methods to benefit from the advantages of each one while producing more reliable and informative predictions.

The softmax output $p(\text {class} \mid \text {image})$ receives it in the deterministic model. A softmax activation function yields probabilities from raw output logits of a neural network. This is crucial to have a proper probability distribution over classes (which addresses aleatoric uncertainty but not epistemic). On the other hand, epistemic uncertainty, which results from a lack of knowledge in the model, can be reduced by providing additional data or building better models. In the deterministic model, dropout is never used in testing (to avoid adding new randomness) and thus network structure remains constant.

### Uncertainty quantification

Predictive modeling will always have a form of uncertainty and uncertain quantification gives us the confidence level in the model. In Bayesian modeling, we usually think of two types of uncertainties:

1. Aleatoric Uncertainty.
2. Epistemic Uncertainty.

DL algorithms are known for high-quality predictions but can often fail to provide a clear picture of the uncertainty inherent in the model. Fortunately, BDL opens the gates for that and enables the network to represent some of its uncertainty available in outputs. Bayesian uncertainty estimates when the network is unsure about a prediction, it can explain its doubts using Bayesian notions. To handle this, a Bayesian DenseNet-121 CNN model is offered for DR classification.

To evaluate how well-calibrated our Bayesian DenseNet-121 CNN model is, we experimented with a few Bayesian approximation methods (MC-Dropout & MFVI and Deterministic) on our pre-processed dataset. To measure uncertainty in Bayesian modeling, we evaluate the posterior distribution of the weights from a neural network using the process outlined in Eq. 2, which approximates the predictive probability for class $c$ given the input $x^*$ and dataset ${\mathcal {D}}$. This approximation is further refined using Eq. 3, which averages the predictions from $T$ recurrent MC dropout samples, yielding a more accurate estimation.

2 $$\begin{aligned} & p(y = c|x^*, {\mathcal {D}}) \approx \int p(y = c|x^*, w)q_{\theta }(w)dw \end{aligned}$$

3 $$\begin{aligned} & \approx \frac{1}{T} \sum _{t=1}^{T} p(y = c|x^*, w^t) \end{aligned}$$

4 $$\begin{aligned} & \approx \frac{1}{T} \sum _{t=1}^{T} p_c^t = p_c \end{aligned}$$

For $w^t$ a predicted weight vector, and *c* the true class. Furthermore, Eq. 4 calculates the final classifier that gives the class with the highest mean prediction probability. When making a prediction, for each input to the trained neural network, we will now also predict $T$ times using MC-dropout.

The total variance and predictive entropy optimize the evaluation of Bayesian CNN’s uncertainty. We measure the average amount of information carried by this predictive and we use what is called a risk, which corresponds to an uncertainty measure in this case known as a predictive entropy. We compute the predictive entropy as a metric of uncertainty which provides an estimate of how much information the entire distribution contains, as shown in Eq. 5:

5 $$\begin{aligned} H_p(y|x^*) = - \sum _{c} p_c \log p_c \end{aligned}$$

Predictive entropy $H_p$ can be normalized to lie between 0 and 1 by dividing by $\log 2C$, as shown in Eq. 6.

6 $$\begin{aligned} H^*_p(y|x^*) = - \sum _{c} p_c \frac{\log p_c}{\log 2C} \end{aligned}$$

We used the AUC and accuracy indicators to assess the capabilities of our Bayesian DenseNet-121 CNN. First, we used predictive entropy histograms to visualize and quantify our models’ prediction uncertainty. These histograms describe the distribution of predictive entropy values, as this is a measure that reflects to what extent the model was confident in its image prediction. The high number of predictive entropy will result in high uncertainty, while the low number will clearly indicate a confident prediction. The combination of AUC, accuracy, and predictive entropy histograms enables the full assessment of our models’ predictive performance and uncertainty quantification.

In this work, our proposed method to embed uncertainty estimates for DL models in a consistent manner such that the neural network can convey uncertainties during their predictions. That facility can be really helpful in medical applications; for example, during DR classification, uncertainty should also be quantified to assist clinical decisions. Experiments in section will show that the proposed strategy is effective.

### Evaluating metrics

We can see the result of our developed models using some metrics like accuracy, recall, precision, and F1-score. This is how we are measuring our predictions against the true value. Now, this is part of the Classification report, but let’s also look at the normalized confusion matrix to get a feel for how it looks in the case of this model training. At last, we check how effective our model is in terms of classification display by plotting a receiver operating characteristic (ROC) curve and AUC. The ROC curve gives a visual display of the trade-off between true positive and false positive, whereas the AUC value provides the aggregate performance across all classification thresholds.

To find the uncertainty in predictions from our Bayesian DenseNet-121 CNN models, we use mean standard deviation and predictive entropy. It is a way to measure the uncertainty of model-predicted values due to their randomness. It is a value between 0 and 1 representing how much uncertainty or disorder lies within the dataset with respect to the number of categories. Our objective is to minimize uncertainty while keeping entropy low with this model.

We use these evaluation metrics as a benchmark for evaluating the performance of models we developed in this work. Performance such as true positive, false negative, and accuracy like sensitivity, specificity, accuracy, and F1-score, which provide key observations about the performance of the model, were derived from these measures.

The formulas used for our assessment measures are expressed in Eqs. 7, 8, 9, and 10:

7 $$\begin{aligned} \text {Accuracy}&= \frac{TP + TN}{TP + TN + FP + FN} \end{aligned}$$

8 $$\begin{aligned} \text {Precision}&= \frac{TP}{TP + FP} \end{aligned}$$

9 $$\begin{aligned} \text {Recall}&= \frac{TP}{TP + FN} \end{aligned}$$

10 $$\begin{aligned} \text {F1 Score}&= \frac{2 \times \text {Precision} \times \text {Recall}}{\text {Precision} + \text {Recall}} \end{aligned}$$

Where:

- TP: True Positive
- TN: True Negative
- FP: False Positive
- FN: False Negative

## Results and discussion

In this section, we present the results of our experimental evaluation of a proposed strategy and study its effectiveness. We also aimed to find areas where improvement or further study could be done. In this paper, we look into various aspects of the algorithm’s performance and provide a detailed analysis of our results through an extensive set of experiments.

**Fig. 5:**
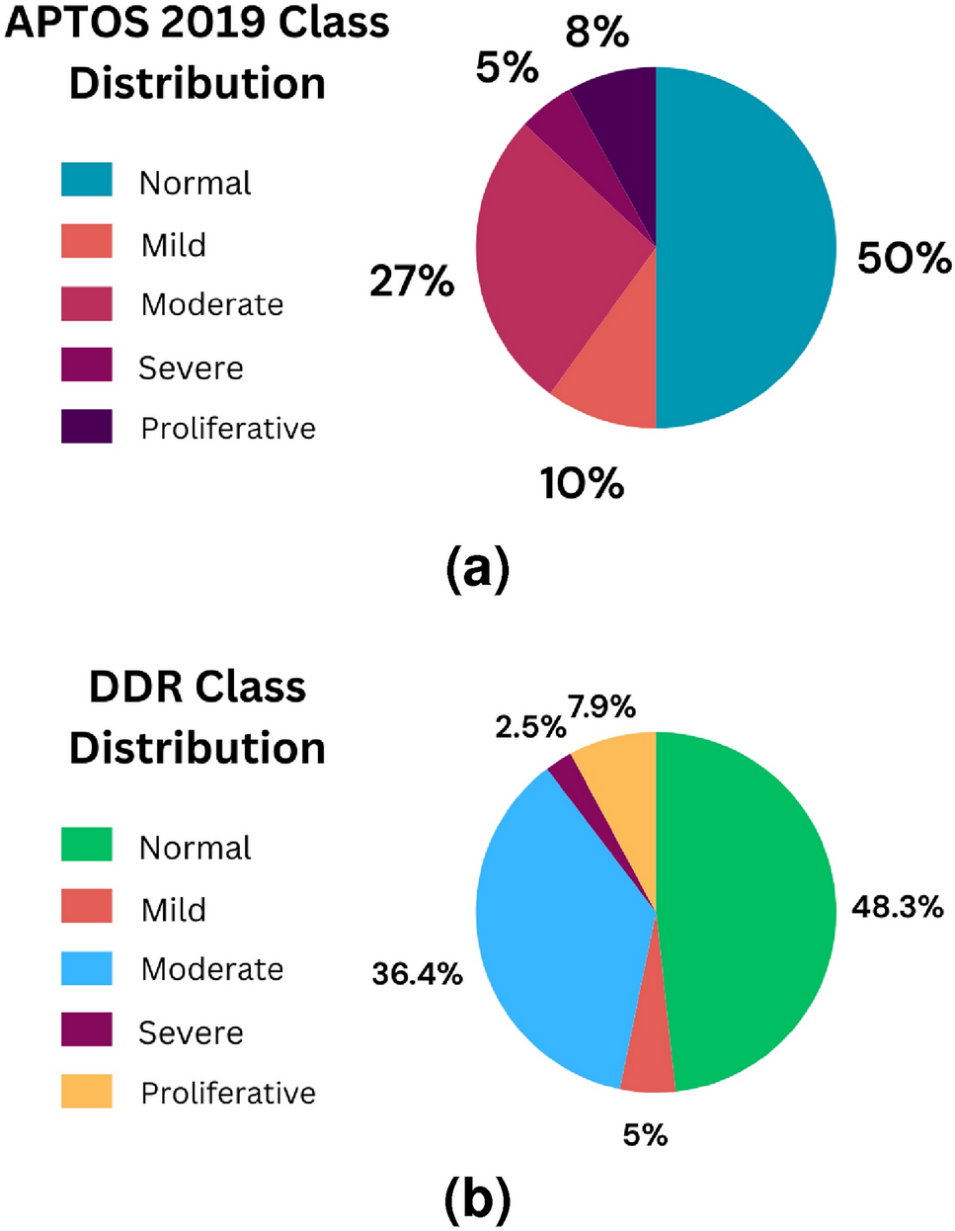
Classes distribution of datasets (APTOS-2019 & DDR).

**Fig. 6:**
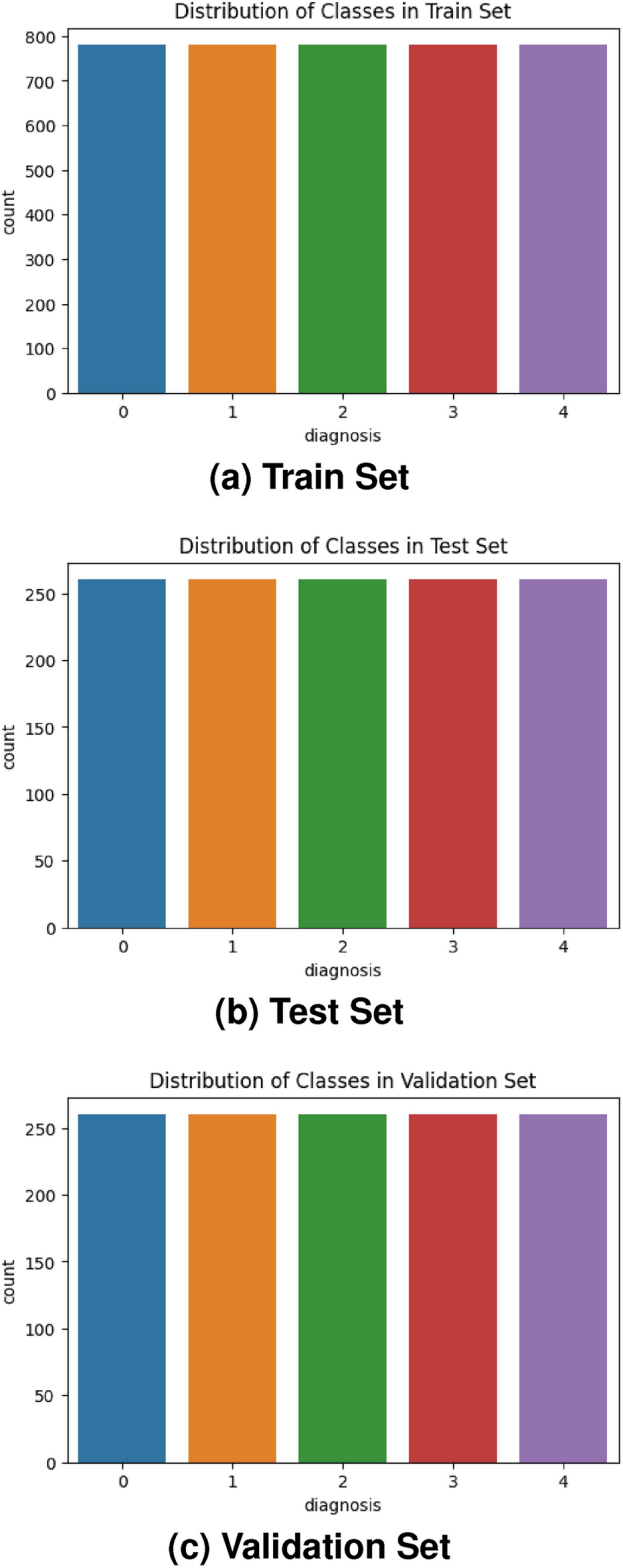
Number of samples in the pre-processed dataset: (**a**) Train set, (**b**) Test set, (**c**) Validation set.

### Data pre-processing

#### Datasets description

We started with Kaggle DR competition datasets APTOS-2019^26^ and DDR^27^, both containing retina images. The files are the retinal fundus samples representing five classes which include No-DR, Mild-DR, Moderate-DR, Severe-DR, and Proliferative-DR. Both datasets are significantly imbalanced, as shown in Fig. 5. The samples were shot in numerous rural areas and under different circumstances, leading them to be not as uniform. As a result, the images had to be pre-processed before being fed into the models. To improve quality, samples were normalized and enhanced using various pre-processing techniques before we could analyze and classify them properly.

**Fig. 7:**
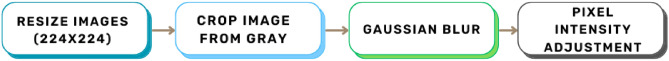
Flow of pre-processing steps applied to the dataset.

Since class imbalance is an issue, we concatenated the samples from both datasets to address it. We balanced the dataset by reducing samples in the majority classes and adding samples in the minority classes from the DDR dataset. This approach helped achieve a more even sample distribution across the classes. This ensured a less biased representation for each class, which in turn facilitated appropriate training and evaluation of these models. We then divided our pre-processed dataset with a ratio of 60-40% to form the train, test, and validation sets with balanced samples of each class, as shown in Fig. 6. Figure 7 illustrates the overall workflow and techniques used during pre-processing.

#### Image pre-processing

In this research, the main pre-processing method is Brightness Enhancement Normalization (BEN)^35^. We aimed to enhance DR diagnosis systems based on DL that target fundus images, using BEN to adjust brightness and contrast. The findings of our study show that applying BEN pre-processing was advantageous for increasing sensitivity, specificity, and overall accuracy compared to strategies without pre-processing or using more common techniques during DR detection algorithms.

The pre-processing steps employed in this study are:

*Image Resizing*: The samples in our merged dataset have different collecting sites and thus their sizes differ greatly. The input sizes for images were scaled uniformly to 224x224. This resize ensured that all images were of equal height and width, simplifying our evaluation efforts.

*Gray Area Cropping (Removing Dark Regions)*: Fundus images often contain large dark areas around the retina, which can add noise. We used a custom cropping function that converts each RGB image to grayscale and applies a threshold of 7 to isolate the retina. This binary mask kept only the central retinal area. If an image was too dark to isolate details, we kept the original to avoid data loss.

*Padding for Uniform Size*: After cropping, some images no longer met the 224x224 target size. We calculated padding values to center the cropped retina in a 224x224 frame, keeping the size uniform without distorting the main retina area.

*Gaussian Blur for Smoothing*: Gaussian blur is applied to each image to reduce pixel variations and smooth out fine details, which allows main retinal features to stand out. We used a standard deviation of 40 to achieve balanced noise reduction and retain important details.

*Brightness and Contrast Adjustment (BEN)*: Finally, BEN applied brightness and contrast normalization to all images: brightness was multiplied by a factor of 4, and contrast was balanced by subtracting a blurred version of the image from itself. This step enhanced DR-specific retinal features while minimizing brightness variations, resulting in images with uniformly highlighted structures.

**Fig. 8:**
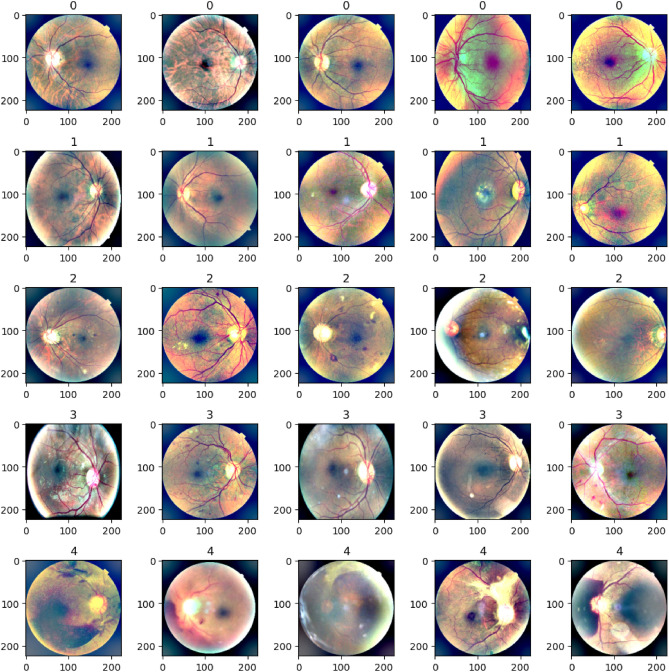
Pre-processed images after applying pre-processing techniques.

This pre-processing created equal input samples for the models and helped to increase the performance of the system as a whole. The pre-processed samples after applying every pre-processing technique are shown in Fig. 8.

### Fine-tuning and training of BCNN models

Bayesian DenseNet-121 CNN model training starts from a pre-trained version of the original DenseNet-121 architecture, utilizing Bayesian approximation techniques like MC dropout and MFVI, as well as deterministic methods.

The BCNN-MC Dropout model was trained with a dropout rate of 0.2-0.5 applied across all layers for 100 epochs. It used the Adam optimizer with a learning rate of 0.00005 and a batch size of 8 (higher batch sizes were constrained by the available GPU RAM). A categorical cross-entropy loss function was chosen based on the probability distribution in its output.

The BCNN-MFVI model was trained for 60 epochs. The optimizer used was Adam, with a learning rate 1e-3 and a batch size of 8. MFVI was employed to fit the mean-field model using a negative log-likelihood loss function. Additionally, a deterministic model was trained for 100 epochs, using the Adam optimizer with a learning rate of 0.0005 and a batch size of 8. The loss function used was categorical cross-entropy. Table 2 provides details on the hyperparameters used.

We used various data augmentation methods to improve representation in underrepresented classes during the model’s training. Techniques like horizontal and vertical flips, rotations, brightness adjustments, and zooming increased the diversity of training data, which strengthened the model’s generalization across all classes. The output layer of the Bayesian DenseNet-121 CNN model comprised a convolutional layer with a softmax activation function to classify the stages of DR.

**Table 2:** Hyperparameter details of the proposed BCNN models.

Hyper-Parameters	BCNN-MC Dropout	BCNN-MFVI	BCNN-Deterministic
Batch Size	8	8	8
Initial Learning Rate	0.001	0.0001	0.001
Maximum Learning Rate	0.00005	1e-3	0.0005
Epochs	100	60	100
Optimizers	Adam	Adam	Adam
Dropout	0.2-0.5	0.2-0.5	0
Activation Function	Softmax and ReLU	ReLU	Softmax and ReLU
Loss Function	Categorical Cross Entropy	Negative log-likelihood	Categorical Cross Entropy

**Fig. 9:**
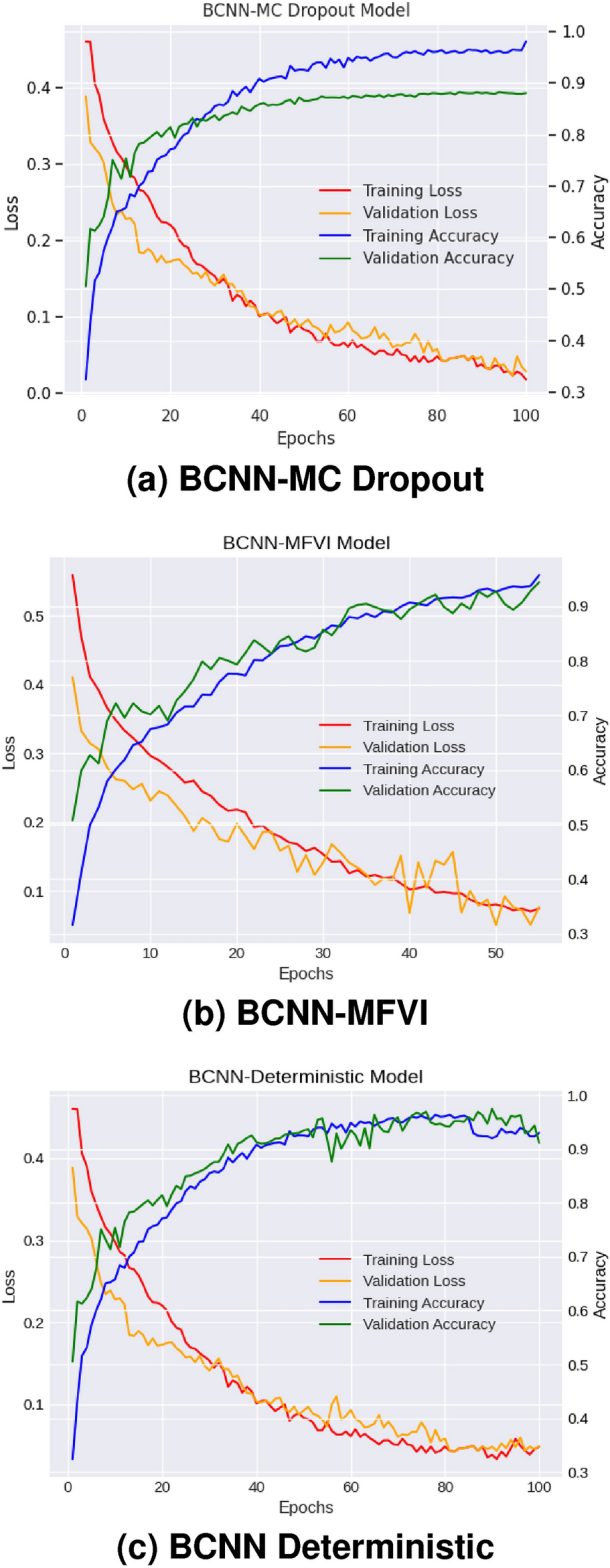
Training accuracy and loss graphs of BCNN models.

### Model performance

This section assesses the performance of our proposed models for DR classification. We evaluated the prediction performance and model uncertainty on a dataset of retinal images and compared the performance of our models against four recent studies on DR classification.

#### Accuracy and loss graphs

The performance of our Bayesian DenseNet-121 CNN models is illustrated in Table 3, which shows the models’ ability to diagnose DR using various metrics, including accuracy, recall, precision, and F1-score on both training and testing datasets. Figure 9 presents the learning curves for accuracy and loss of the Bayesian DenseNet-121 CNN models. The BCNN-MC Dropout model achieved a training accuracy of 98.03% and outperformed the other two BCNN models: the BCNN-MFVI model (training accuracy of 95.70%) and the BCNN-Deterministic (training accuracy of 93.16%). The testing accuracy of the BCNN-MC Dropout model was 97.68%, with precision, recall, and F1-score all at 97%. In comparison, the BCNN-MFVI model achieved a testing accuracy of 94.23%, with precision at 94%, recall at 93%, and F1-score at 94%, while the BCNN-Deterministic model achieved a testing accuracy of 91.44%, with precision at 92%, recall at 91%, and F1-score at 91%. These data show that the BCNN-MC Dropout model has a higher confidence level regarding recall and F1-score, implying better DR classification regardless of the type of DR.

**Table 3:** Performance of proposed Bayesian DenseNet-121 CNN models for DR Classification.

Model	Train accuracy (%)	Train loss	Test accuracy (%)	Test loss	Precision (%)	Recall (%)	F1-Score (%)
BCNN-MC Dropout	98.03	0.018	97.68	0.08	0.97	0.97	0.97
BCNN-MFVI	95.70	0.074	94.23	0.086	0.94	0.93	0.94
BCNN-Deterministic	93.16	0.048	91.44	0.10	0.92	0.91	0.91

#### Confusion matrices

**Fig. 10:**
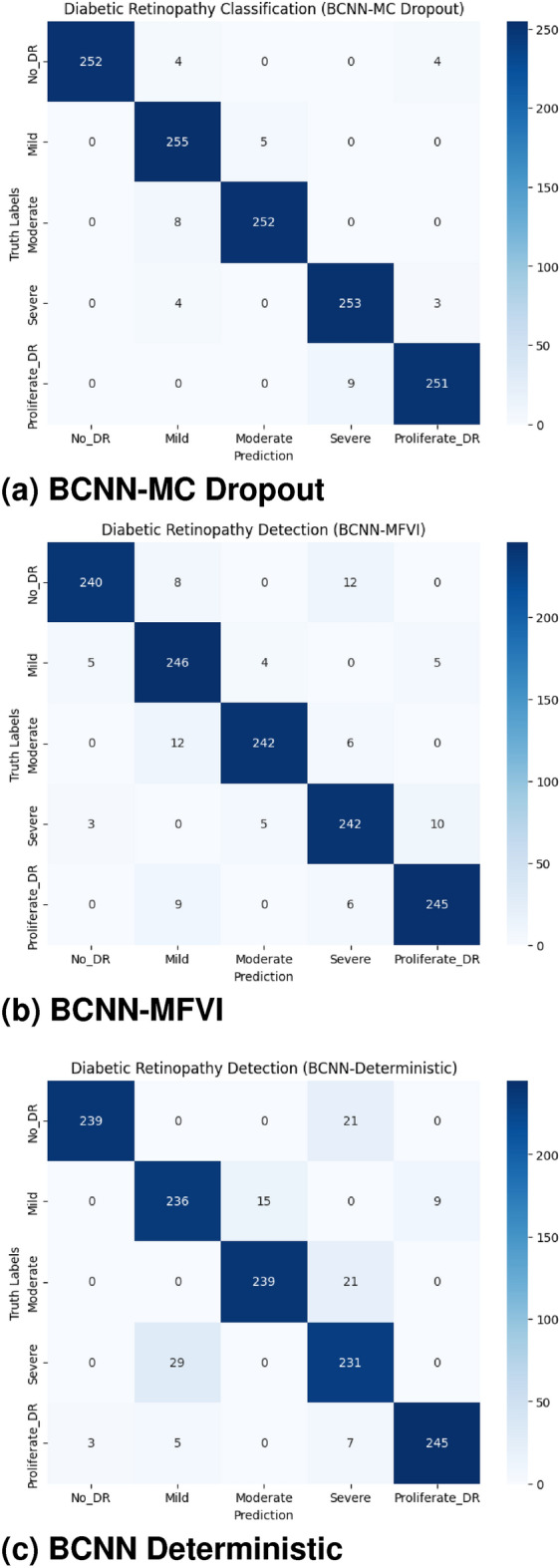
Confusion matrices of BCNN models on test data. .

We also used confusion matrices to evaluate the performance of our BCNN models more deeply, as presented in Fig. 10. For the multi-class classification test set, the BCNN-Deterministic model displayed slightly more false negatives and false positives (compared to BCNN-MFVI and BCNN-MC Dropout models) on both test sets. The BCNN-MC Dropout model, in particular, showed lower false negative (FN) and false positive (FP) rates. Figure 10a demonstrates that each severity level achieves the highest rates of correct classification. In contrast, Fig. 10b shows that the No-DR category had poor correct classification among all severity levels. Similarly, in Fig. 10c, the Severe class had a lower accuracy rate compared to other severity levels. These observations indicate that the BCNN-MC Dropout model generalizes best on unseen data.

**Fig. 11:**
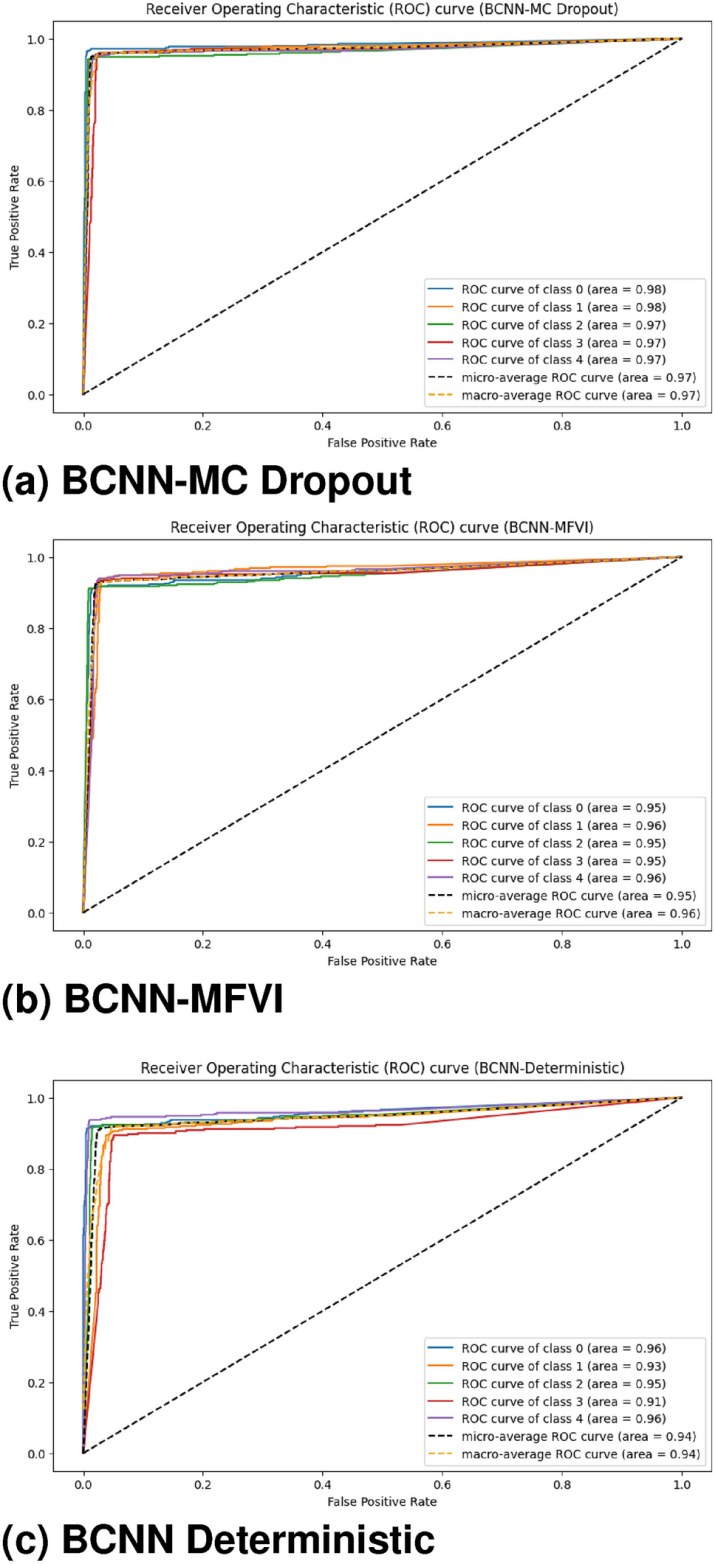
ROC curves of the BCNN models on test data. .

#### Receiver operating characteristic (ROC) curves

Figure 11 presents the ROC curves for DR classifications. These curves visually depict the model’s classification performance, with the AUC indicating the area under the curve. For the BCNN-MC Dropout model, No-DR and Mild DR achieved the highest AUC values (98%), followed by Moderate, Severe, and Proliferative DR, each with 97% AUC. In the BCNN-MFVI model, Mild DR and Proliferative DR had the highest AUC values (96%), followed by No-DR (95%), with Moderate and Severe DR each scoring 85%. The BCNN-Deterministic model performed best with No-DR (96%) and Proliferative DR (96%), followed by Mild DR (93%), and Moderate and Severe DR with average AUCs. These results suggest that the BCNN-MC Dropout model is the most confident, as indicated by its Bayesian posterior probability distribution. Higher AUC values represent better predictive performance, confirming the models’ effectiveness in DR classification.

#### Uncertainty quantification

Measuring uncertainty is important for a model to be reliable in high-stakes tasks like DR detection, where it has been shown that data variability can affect predictions. Using predictive entropy and variance estimation, our proposed Bayesian DenseNet-121 model can represent uncertainty that improves the accuracy and robustness of decision-making.

*Predictive Entropy*: Predictive entropy indicates the confidence of the model across classes and gives us an idea of how certain a prediction was. A lot of uncertainty equals high entropy, which is useful for identifying low-confidence cases that the clinician may want to take a closer look at. The predictive entropy $H_{\text {pred}}(y|x)$ for an input $x$ is computed as:

11 $$\begin{aligned} H_{\text {pred}}(y|x) = - \sum _{c} p(y = c|x) \log p(y = c|x) \end{aligned}$$

where the summation spans all classes $c \in \{0, 1, 2, 3, 4\}$. This says how the confidence is distributed among possible classes. Low values of entropy make the model predictions more confident, while high values signify uncertainty which could potentially allow for a review in clinical deposit.

*BCNN-MC Dropout*: The BCNN-MC Dropout model performs Monte Carlo (MC) Dropout during inference as an approximation to the posterior. MC Dropout provides estimates of both aleatoric and epistemic uncertainty by taking the average over multiple stochastic passes. This mitigates overconfidence when uncertainty is high, an important property in medical imaging. For each input $x$, the predictive probability $p(y = c | x)$ is estimated as follows, using $T$ stochastic forward passes:

12 $$\begin{aligned} p(y = c \mid x) \approx \frac{1}{T} \sum _{t=1}^T p_\theta (y = c \mid x, {\hat{w}}_t), \end{aligned}$$

where ${\hat{w}}_t$ are the weights sampled under dropout. This ensemble average derives due to the robustness of predictions in uncertain cases. We compute the variance across predictions (predictive uncertainty) as follows:

13 $$\begin{aligned} \sigma ^2(y|x) \approx \frac{1}{T} \sum _{t=1}^T \left( p(y|x, {\hat{w}}_t) - {\bar{p}}(y|x) \right) ^2, \end{aligned}$$

where ${\bar{p}}(y|x)$ is the mean predictive probability over all samples. This variance measure prevents overfitting by reducing the confident predictions of the model against noisy data. Using Bayesian optimization to choose between dropout rates (0.2-0.5) and regularization (L2, $5 \times 10^{-5}$), we found that increasing flexibility in model capacity was beneficial as long as it did not lead to excessive overfitting.

*BCNN-Mean Field Variational Inference (MFVI)*: The BCNN-MFVI model approximates a posterior distribution of the model weights with MFVI. For example, MFVI uses an index of fully factorized Gaussian posterior. It is efficient as it allows fast calculation of the Evidence Lower Bound (ELBO) to trade off accuracy and regularization, preventing regretfully overconfident predictions. This translates to better generalization through model uncertainty compensation:

14 $$\begin{aligned} q(w) = \prod _{i=1}^N {\mathcal {N}}(w_i; \mu _i, \sigma _i^2) \end{aligned}$$

The model is optimized by minimizing the ELBO:

15 $$\begin{aligned} \text {ELBO} = {\mathbb {E}}_{q(w)}[\log p(D|w)] - \text {KL}(q(w) \Vert p(w)), \end{aligned}$$

where $\text {KL}$ is the Kullback-Leibler divergence of the posterior $q(w)$ and prior $p(w)$. This kind of optimization leads the model to generalization since it is a trade-off between data likelihood and model complexity, thus enhancing robustness.

*Deterministic Baseline (BCNN-Deterministic)*: We also implemented a baseline (BCNN-Deterministic) model for comparison. This model has the same architecture as our newly developed BCNN-MC Dropout except no stochastic inference is modeled, and output probability is estimated by softmax. The softmax probabilities for class $i$ and input $x$ are calculated as:

16 $$\begin{aligned} p(\text {class} = i \mid \text {image} = x) = \frac{e^{z_i}}{\sum _{j} e^{z_j}}, \end{aligned}$$

where $z_i$ is the logit for class $i$. This setup captures aleatoric uncertainty; however, it lacks the Bayesian inference to include epistemic uncertainty and thus is less robust compared to Bayesian models.

#### Uncertainty comparison using predictive entropy

**Fig. 12:**
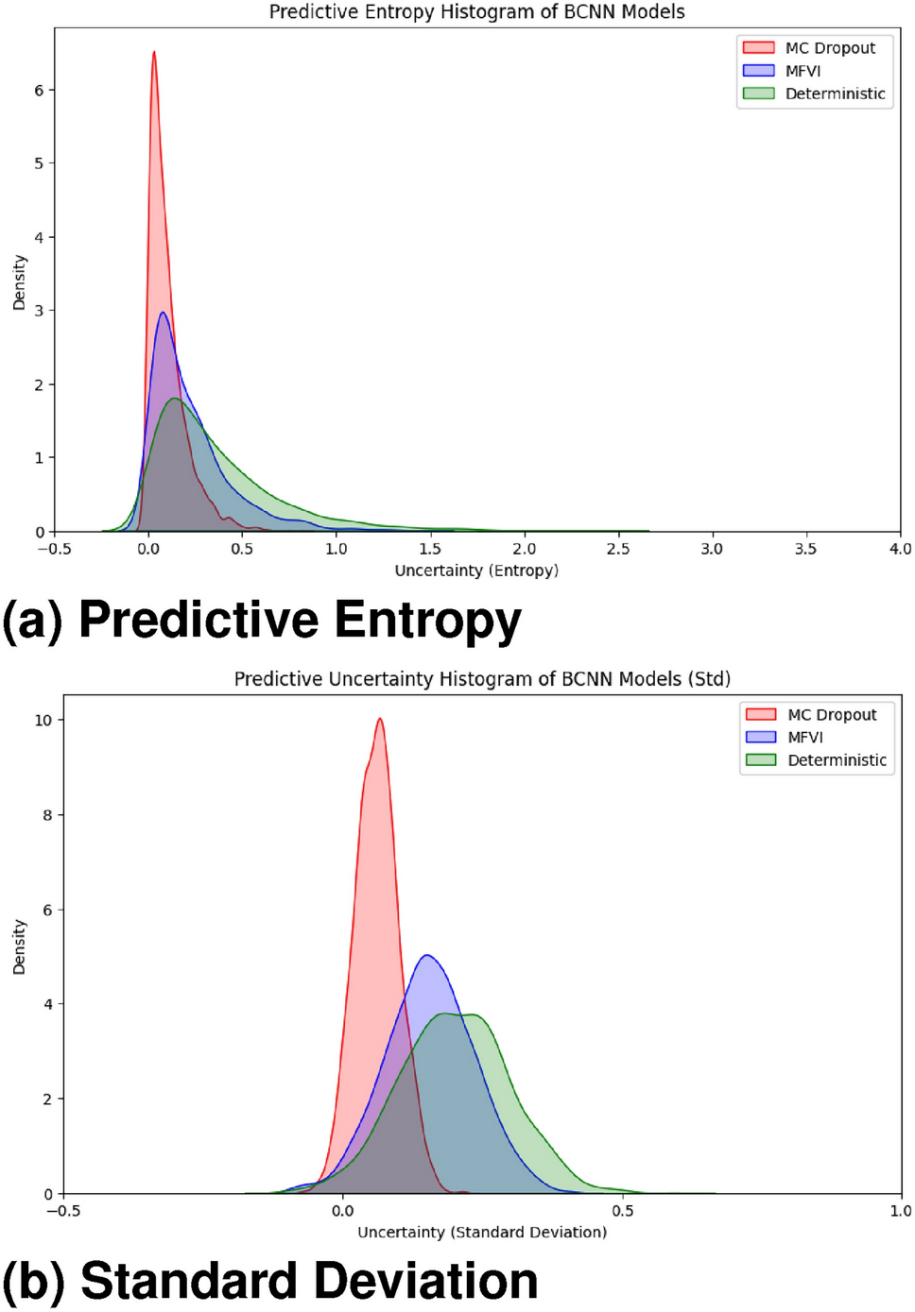
Uncertainty distribution for BCNN model predictions.

We utilize the predictive entropy for quantitative measurement of model confidence between different models. We compute the entropy for a particular output distribution, as follows:

17 $$\begin{aligned} H(p) = -\sum _{i=1}^5 p_i \log (p_i), \end{aligned}$$

where $p_i$ is the probability of a sample belonging to class $i$. That lower entropy means confident predictions. Compared with BCNN-MFVI and the deterministic model, Fig. 12 shows that the predictive entropy of BCNN-MC Dropout is lower while being a more valid predictor. The results support the superiority of the Bayesian DR detection model over others in terms of managing and representing uncertainty, thus confirming that our approach allows predicting with more robustness and accuracy regarding DR detection.

**Fig. 13:**
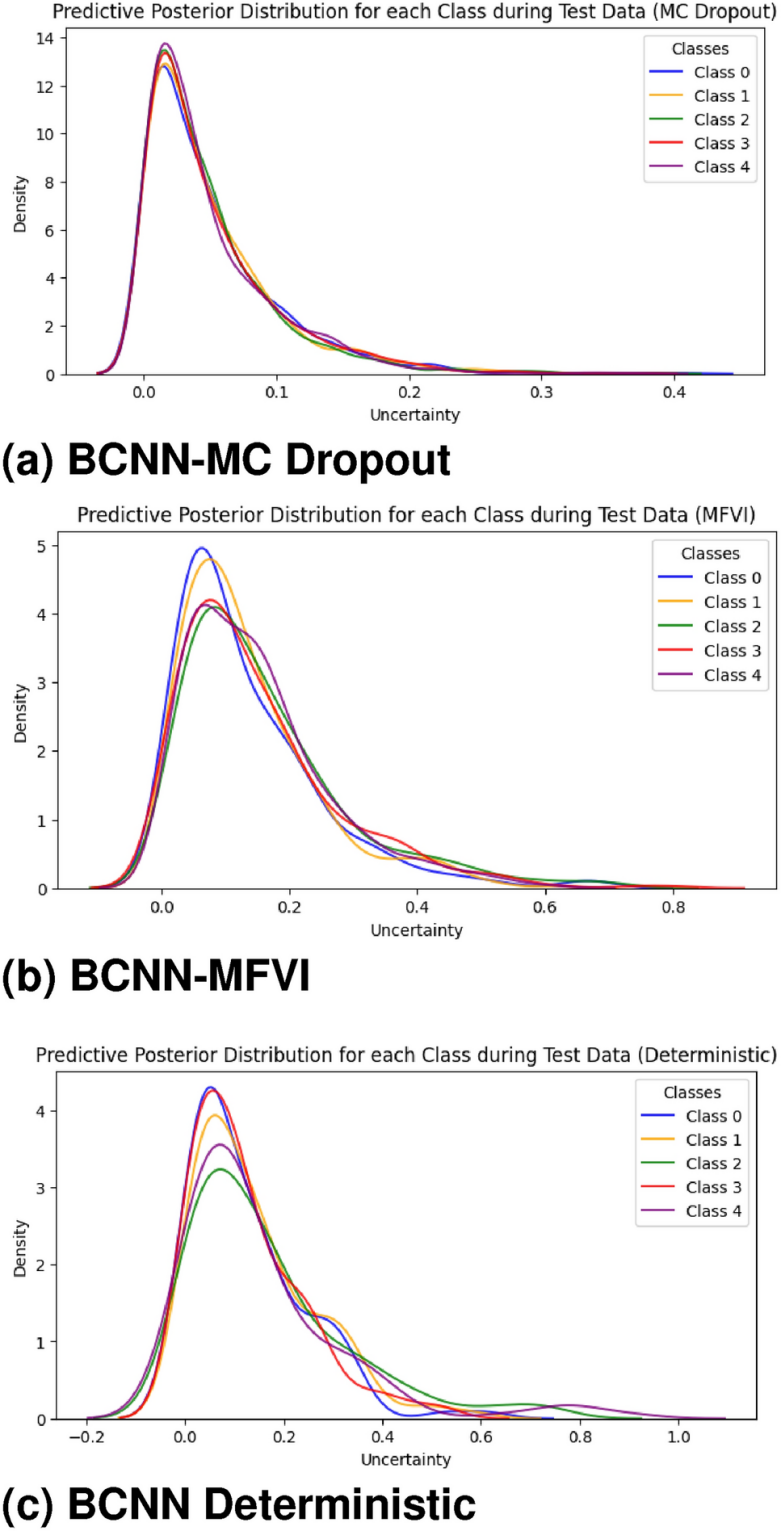
Predictive posterior distribution of BCNN models for each class on the test set.

In Fig. 13, we can visualize the uncertainty for each class predicted by our models. Class 4 (Proliferative-DR) shows the lowest uncertainty for the BCNN-MC Dropout model compared to other classes. For instance, class 0 (No-DR) has the minimum uncertainty for the BCNN-MFVI model, and class 1 (Mild) shows the least uncertainty for the BCNN deterministic baseline. This analysis enables us to convert uncertain classification results into expert-like uncertainty bounds, which can be highly beneficial in medical applications. Although this approach is still in its early stages, the implementation of BNNs could provide significant advantages when integrated into diagnostic systems as part of the clinical workflow. Future work could explore alternative uncertainty quantification approaches using ensemble models and more advanced Bayesian inference techniques to further evaluate performance in this specific environment.

**Fig. 14:**
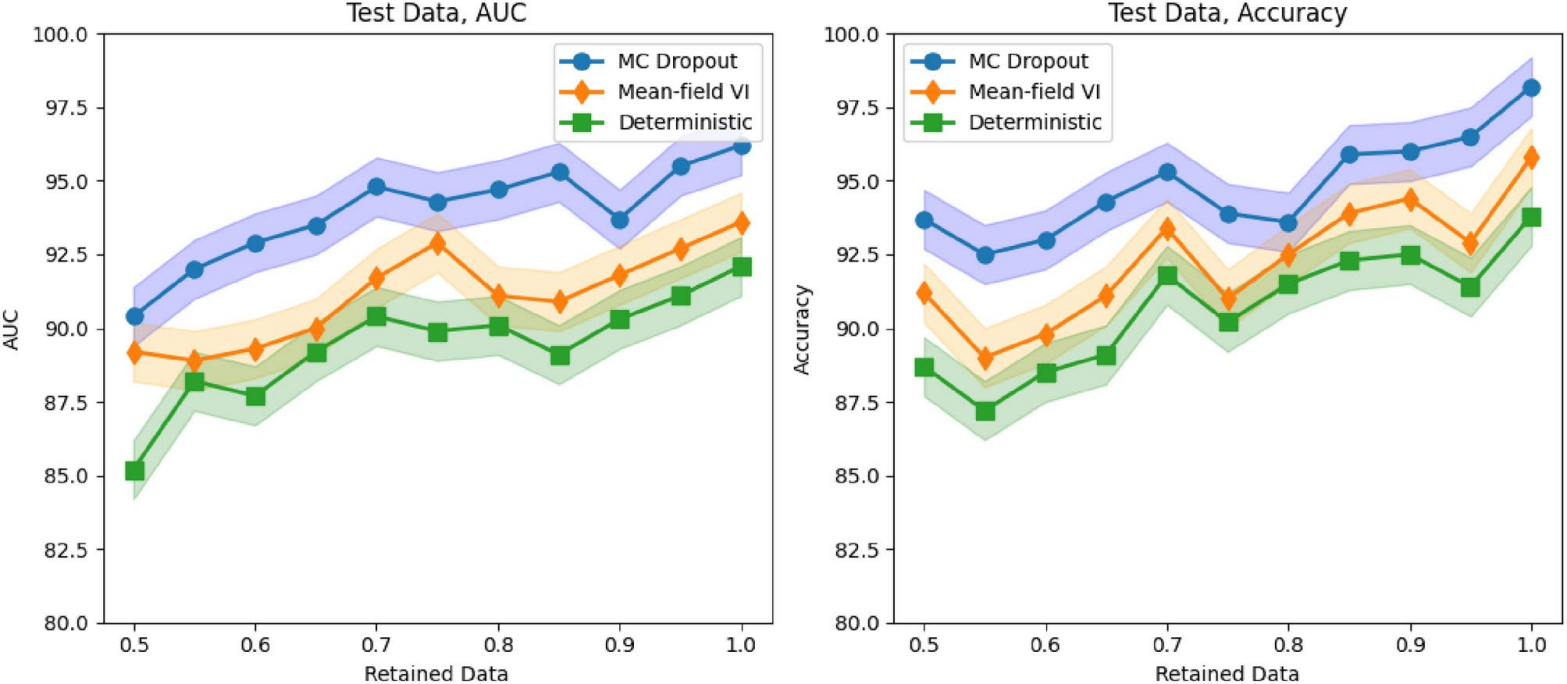
In-distribution performance of BCNN models.

Additionally, we tested our models with different data retention levels and visualized the performance of BCNN models in terms of AUC and Accuracy across these levels. Table 4 and Fig. 14 illustrate the quantitative performance of various approaches as the amount of retained data increases. The methods that effectively capture meaningful uncertainty estimates demonstrate improved performance (greater AUC and accuracy) as the referral rate decreases (or the retained data increases). Specifically, methods with steeper slopes in the graphs better estimate uncertainty, as they systematically refer to data points where their predictions are less reliable. This consistency supports our argument that the BCNN-MC Dropout model achieves the most significant performance gain, followed by improvements in the BCNN-MFVI model, which in turn outperforms the deterministic BCNN method. The performance of all methods converges as more data is retained, indicating that the models perform similarly well overall, allowing for a fair comparison of their uncertainty estimation capabilities.

**Table 4:** Performance of Bayesian Convolutional Neural Network (BCNN) models (AUC and Accuracy) with varying amounts of data retained.

Method	50% data retained		70% data retained		100% data retained	
	AUC $\uparrow$	Accuracy $\uparrow$	AUC $\uparrow$	Accuracy $\uparrow$	AUC $\uparrow$	Accuracy $\uparrow$
MC Dropout	90.4 ± 0.8	93.7 ± 0.3	94.8 ± 0.6	95.3 ± 0.3	96.2 ± 0.2	98.2 ± 0.1
Mean-field VI	89.2 ± 1.0	91.3 ± 0.4	91.7 ± 0.8	93.4 ± 0.4	93.6 ± 0.8	95.8 ± 0.3
Deterministic	85.2 ± 0.7	88.7 ± 0.6	90.4 ± 0.9	91.8 ± 0.8	92.1 ± 0.3	93.8 ± 0.2

These overall results indicate that the advantage of using Bayesian models lies in their ability to identify when they are uncertain and indicate when their predictions may be unreliable. A model that can effectively handle uncertainty is crucial for applications in medicine, where models assist in diagnoses and other clinical decisions.

### Models robustness analysis

**Fig. 15:**
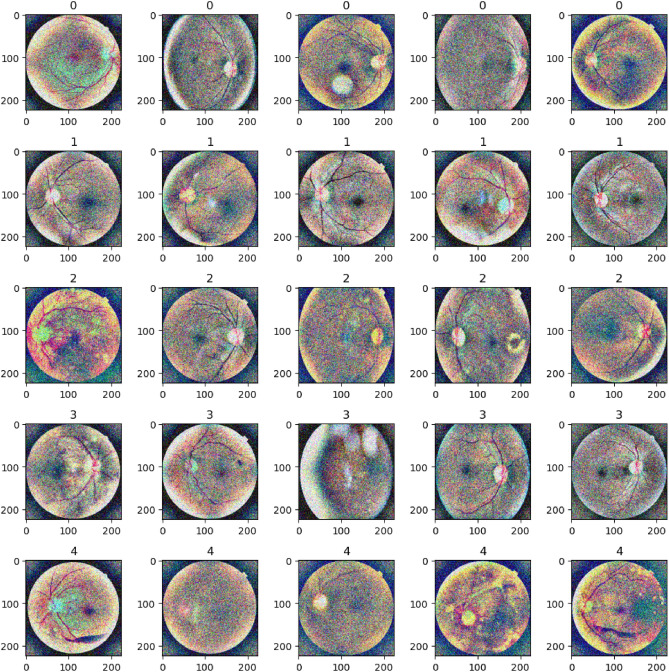
Noise injection on the test data samples.

In this section, we verify the robustness of our proposed models by injecting noise into the testing set. This is important in the medical domain because in medical domain, data always come in noise form, so models must be robust so that they also predict accurately the noise data. We have added Gaussian noise to assess model robustness against noisy DR samples. Once the images are pre-processed normally (resized, converted to RGB, and contrast-enhanced), we add Gaussian noise based on mean and standard deviation, details of which will decide the noise intensity level. It is constantly added on top of the processed pixel values, bringing random deviations to represent real-world variations or imperfections in the image acquisition process. After that, we clip the pixel values in the range of 0-255. The noise injection helps us simulate noisy data to see the model’s robustness in case the images contain noisy data. Figure 15 shows the test data samples on which we inject the noise in test data to test the model’s robustness.

**Fig. 16:**
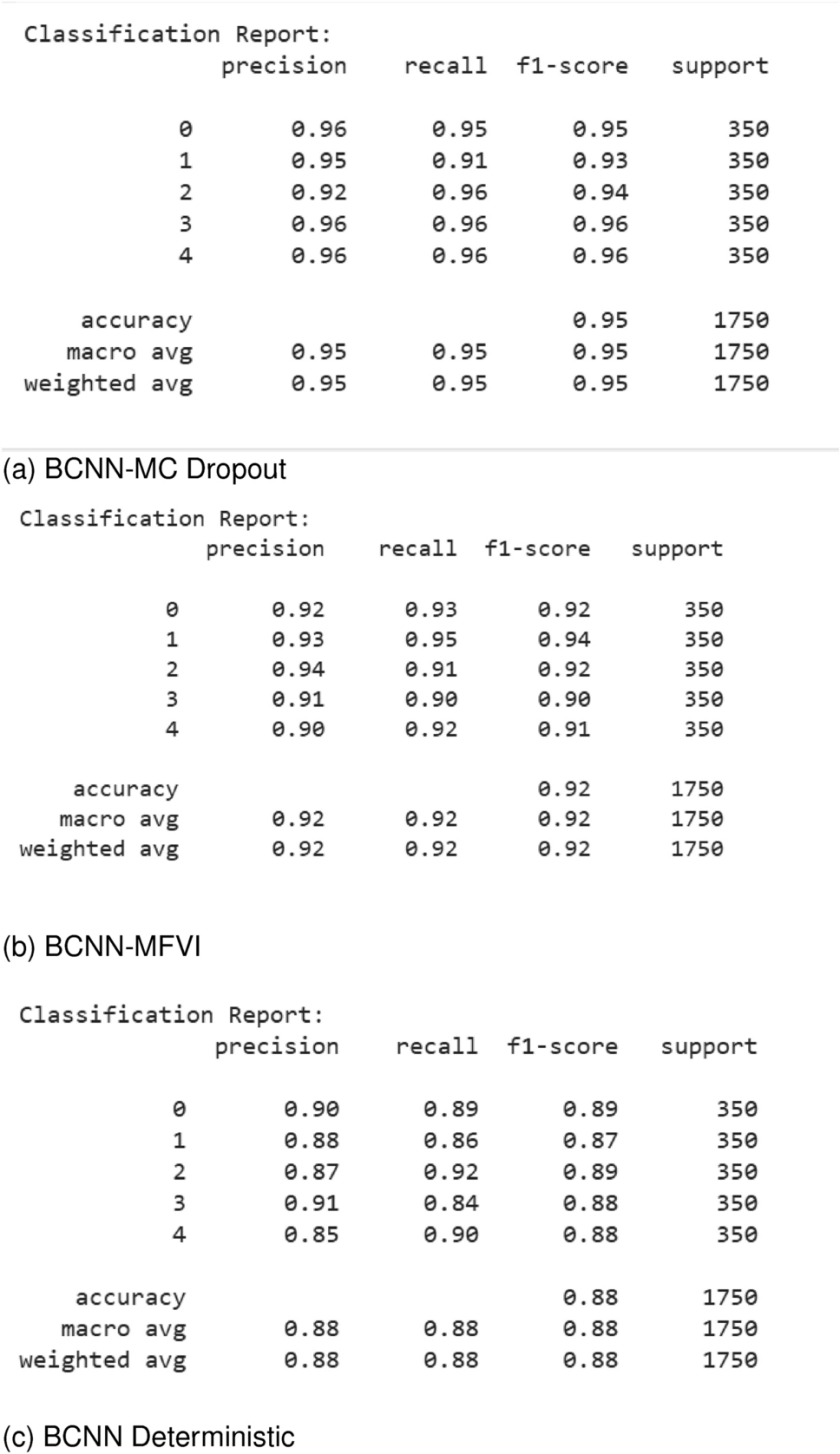
Classification reports of proposed models after testing on noisy test samples.

After adding the noise at different intensity levels, we test the models on the noisy data and evaluate the models’ performance using key metrics like accuracy, precision, recall, and F1-score. Here are the classification reports of each of our models after testing on noisy data to validate the robustness of the models. Figure 16 shows that our model BCNN-MC Dropout outperforms the other models by performing constantly well even in the noisy test data, which shows the robustness of our model. Other models like BCNN-MFVI and BCNN-Deterministic also perform well under noisy test samples.

### Comparison with state-of-the-art studies

In this section, we compare the performance of our proposed method with recent state-of-the-art studies on DR datasets. The results for different models on the DR dataset images, as used in our comparison, are shown in Table 5. Our results emphasize that the proposed BCNN-MC Dropout model has surpassed all previously reported studies, achieving a training accuracy of 98.03% and a testing accuracy of 97.68%, thereby surpassing the accuracies reported in other studies. This empirically proves that our proposed BCNN-MC Dropout model outperforms existing models in the literature.

**Table 5:** Comparison of suggested models with existing DR classification research.

Reference	Year	Method	Accuracy
Filos et al.^36^	2019	Ensemble MC-Dropout MFVI	87.1% 81.1%
Nguyen et al.^37^	2020	VGG-16 VGG-19	80% 82%
Taufiqurrahman et al.^38^	2020	MobileNetV2-SVM	85%
Yi et al.^39^	2021	Residual Attention EfficientNet	93.5%
Gangwar & Ravi^40^	2021	Hybrid Inception-ResNet-v2	82.18%
Islam et al.^41^	2022	SCL (Supervised Contrastive Learning)	84%
Alahmadi^42^	2022	Recalibration of style and content by DL	85%
Oulhadj et al.^43^	2022	DenseNet, InceptionV3, ResNet-50	85.28%
Butt et al.^44^	2022	ResNet-18 GoogleNet	89.29%
A.M. Fayyaz et al.^45^	2023	AlexNet ResNet-101	93.0%
Tiwari^46^	2023	ResNet50	91.60%
W. K. Wong^47^	2023	ShuffleNet ResNet-18	82% 75%
A. Jabbar^48^	2024	GoogleNet + ResNet	94%
Current Work	–	BCNN-MC Dropout BCNN-MFVI BCNN-Deterministic	97.68% 94.23% 91.44%

Furthermore, we conducted experiments using diverse evaluation metrics such as accuracy, precision, recall, F1-score, and AUC in addition to the ROC curve to validate the robustness and efficacy of our methodologies for DR classification. These results are meaningful for medical image analysis and can be applied to improve disease diagnosis and treatment outcomes in clinical practice^9^.

#### Comparison with Non-Bayesian baseline models

The added value of our Bayesian approach for DR detection by comparing it to standard non-Bayesian models^37,43–48^. Our Bayesian DenseNet-121 models (BCNN-MC Dropout, BCNN-MFVI) achieve superior performance over non-Bayesian baselines, including standard DenseNet, ResNet-50, etc., particularly in medical settings where diagnostic accuracy is critical. Table 5 also shows the accuracies of non-Bayesian models as well as our proposed Bayesian models, which clearly represent the effectiveness of the Bayesian approach. Here is the major improvement of the Bayesian model over non-bayesian baseline models:

*Uncertainty Quantification*: Bayesian models provide measures of uncertainty (e.g., predictive distribution entropy) that indicate low-confidence predictions, availability that is not readily present in conventional models, and can help find areas that may need further inspection. This has a special relevance in clinical applications where certainty of prediction directly influences treatment decisions.

*Accuracy improvement*: The Bayesian DenseNet-121 (BCNN-MC Dropout) achieved a testing accuracy of 97.68% compared to standard baseline model accuracies^37,43–48^, indicating superior classification performance.

*Robustness to Noise*: Against corrupted images, our Bayesian models maintained higher accuracy even in noisy data than other nonbaseline models as we experimented on noisy data, an essential trait for deployment due to the natural variance in image quality.

## Conclusions and future work

In this work, we aimed to fine-tune current state-of-the-art pre-trained DL models for DR classification using our pre-processed dataset. We also sought to quantify uncertainty in model predictions through Bayesian modeling approximation techniques. We enhanced the DenseNet-121 pre-trained model with Bayesian approximation techniques, including MC-Dropout, MFVI, and deterministic approaches, to develop a Bayesian DenseNet-121 CNN model capable of quantifying uncertainty by analyzing the posterior predictive distribution. Our focus was on effectively modeling uncertainty to detect and classify DR in retinal images.

We experimentally verified our method using a pre-processed dataset. The experimental results provide evidence for the accuracy of our proposed methods, demonstrating the ability to quantify uncertainty, particularly with Bayesian models. Our Bayesian DenseNet-121-based CNN models achieved high accuracy in DR classification while enabling the quantification of uncertainty in classification results, which is crucial in clinical practice.

In our experiments, the BCNN-MC Dropout model achieved a testing accuracy of 97.68%, with the BCNN-MFVI model showing the second-best performance at 94.23% testing accuracy and the BCNN-Deterministic model achieving a testing accuracy of 91.44%. Furthermore, our findings show that our method works just as well as the best current methods at finding DR. This shows that our BCNN models could be useful for medical image analysis tasks.

Future work may involve testing on alternate DR-related datasets and also investigating alternative Bayesian approaches, including ensemble models and more advanced inference techniques for uncertainty quantification and model performance. In the future, more work will be done in these areas to enhance the clinical utility of the model and facilitate better decision-making for medical diagnostics due to proper uncertainty estimates with good calibration.

In conclusion, we have developed a state-of-the-art method in Bayesian models for image analysis and transfer learning using pre-trained models. This study demonstrates the importance of uncertainty quantification in improving disease diagnosis and treatment outcomes and offers guidelines for addressing these issues. By overcoming the challenges and identifying barriers, this work provides insight into modeling uncertainty, which will aid in creating more effective and precise diagnostic tools for DR and other diseases.

## Data Availability

Data is provided within the manuscript.
